# Research on the impact of transceiver isolation of GB-SAR on imaging quality

**DOI:** 10.1371/journal.pone.0360315

**Published:** 2026-10-29

**Authors:** Shanshan Hou, Xiaolin Yang, Kangyu Deng

**Affiliations:** 1 China Academy of Safety Science and Technology, Beijing, China; 2 Cathay Safety Technology Co., Ltd., Beijing, China; 3 College of Civil Engineering, Hunan University, Changsha, Hunan, China; Gulf University for Science and Technology, KUWAIT

## Abstract

Ground-Based Synthetic Aperture Radar (GB-SAR) systems frequently suffer from insufficient transceiver isolation due to their compact architecture. This insufficiency leads to transmitted signal leakage into the receiving channel, generating third-order intermodulation distortion (IM3). Consequently, the imaging signal to noise ratio (SNR) is degraded and artifacts in the range direction are introduced. Utilizing an independently developed Ku-band GB-SAR system termed S-SAR M, this study explores the influence mechanism of transceiver isolation on imaging quality through dynamic adjustment of antenna spatial parameters. It identifies a system-specific critical isolation threshold and an optimal design range, thereby offering a theoretical basis and practical guidelines for hardware optimization of GB-SAR systems. The isolation model is quantitatively calibrated against the measured horn radiation pattern and antenna geometry with a root-mean-square error of 1.8 dB, and the critical threshold is derived analytically from receiver nonlinearity parameters through the third-order input intercept point (IIP3). The results indicate that antenna angle is the dominant factor for isolation improvement, with an average enhancement of 56.3 percent achieved as the angle increases from 0 to 60 degrees. Antenna separation exhibits a synergistic effect on isolation at larger angles. A critical isolation threshold of 74 dB is established to suppress range artifacts induced by IM3 in the S-SAR M system; below this threshold, artifacts consistently occur. Moreover, a tri-segmented nonlinear relationship between isolation and SNR is observed, with an optimal balance region located between 74 dB and 80 dB where the peak SNR is maintained. One-way analysis of variance delivers a result with p less than 0.001 and confirms the significance of these trends. A quantitative artifact-to-background energy ratio further corroborates the visual artifact classification. These findings offer important insights for enhancing the performance of GB-SAR systems. It should be noted that the reported numerical thresholds are specific to the S-SAR M hardware configuration; a normalized isolation-margin framework is therefore provided to transfer the methodology, with platform-specific calibration, to GB-SAR systems using other frequency bands or receiver architectures.

## 1 Introduction

Ground-based Synthetic Aperture Radar (GB-SAR) technology has become a core technology in the fields of slope stability assessment and geological disaster early warning [1–3]; Ground-based SAR interferometry has since matured into a well-documented geodetic technique for surface deformation monitoring [4,5]. It offers sub-millimeter deformation monitoring accuracy and all-weather, all-time observation capabilities. The irreplaceable nature of this technology stems from three core advantages. First, its near-field observation capability enables centimeter-level spatial resolution and minute-level revisit cycles. This feature precisely captures the transient deformation of the slopes. Second, Ku/Ka-band signals have strong penetration through clouds and fog. They ensure continuous monitoring under adverse conditions, such as heavy rain and nighttime. Third, the orbital scanning mechanism supports 120° sector coverage with a maximum monitoring distance of 2 km. This capability meets the large-scale monitoring requirements of major infrastructure projects [6,7]. International engineering practices have demonstrated these benefits. These include landslide monitoring in Elba, Italy, and rock mass stability assessment in the Swiss Alps. GB-SAR can advance the landslide warning window by over 72 h before a disaster. This capability significantly enhances the performance of disaster prevention and control [8,9].

However, the fundamental bottleneck exposed in engineering applications severely limits the release of technical potential. This bottleneck is the insufficient transmit-receive isolation caused by compact system design. To adapt to deployment in complex outdoor terrains, GB-SAR must integrate transmit and receive antennas within a compact orbit of 1–2 m. This arrangement results in limited physical antenna spacing [10,11]. Insufficient isolation between co-located transmit and receive channels has been recognized as the fundamental sensitivity bottleneck of continuous-wave radar since the classic analysis of linear FMCW systems [12]. The design flaw causes significant transmit signal leakage. The leakage intensity can reach −40 dBm at a transmit power of 30 dBm. The strong leakage signals interact with the target echoes in the front-end nonlinear components of the receiver. These components include low-noise amplifiers and mixers. This interaction induces third-order intermodulation distortion (IM3) [13,14]. IM3 distortion triggers a dual degradation effect. On one hand, its broadband noise characteristics increase the equivalent noise figure of the system. This increase results in a measured signal-to-noise ratio (SNR) degradation of 5–15 dB [15,16]. On the other hand, it generates ghost artifacts in the range domain. These artifacts are symmetrical to the real target. The position offset is proportional to the time delay. They severely interfere with target identification and phase estimation accuracy. This issue is particularly prominent in high-frequency systems, such as the Ku/Ka bands. It is a key technical bottleneck hindering the imaging quality and deformation inversion reliability of GB-SAR.

Current international research primarily focuses on two approaches. The first is optimizing the signal processing layer. The second is adjusting the system parameters. The former improves resolution by enhancing imaging algorithms. It also develops adaptive filters to suppress environmental noise. Another technique is using multiphase interferometry to enhance phase stability [17,18]. The latter relies on an expanded bandwidth to improve the range resolution. It may also use an extended integration time to enhance the SNR [19,20]. Despite local progress, these methods have fundamental limitations. None of them address the physical coupling mechanism between the transmit-receive isolation and imaging quality. The theoretical gaps are manifested as follows. First, existing research attributes SNR degradation and artifact errors caused by IM3 distortion to environmental noise or algorithmic flaws. It lacks a comprehensive understanding of the chain from isolation to leakage intensity, then to IM3 distortion power, and finally to imaging degradation. Second, the engineering community relies on empirical values for system design. However, the critical isolation threshold for artifact elimination has never been experimentally established for compact Ku-band GB-SAR systems. This gap leads to design blindness. Finally, existing optimization strategies have dimensional limitations. RF shielding can improve isolation but violates the principle of system compactness. Digital cancellation techniques cannot suppress IM3 distortion and increase computational load. The potential for dynamic coordination of antenna spatial parameters has not been systematically quantified. Additionally, the negative effects of high isolation have been overlooked. High isolation potentially sacrifices beam coverage efficiency. These gaps have placed GB-SAR systems in a dilemma. Compactness and high performance cannot be achieved simultaneously. This situation necessitates the establishment of a quantitative mapping theory between isolation and imaging quality to overcome technical bottlenecks [21–23]. Although antenna theory qualitatively establishes that orientation and spacing govern inter-antenna coupling, the complete chain from isolation through leakage power and IM3 to imaging degradation has never been experimentally quantified for compact Ku-band GB-SAR, which is the specific gap addressed by the present study.

This study quantitatively established and verified, through a rigorous experimental method with strictly controlled variables, that 74 dB is the critical isolation threshold for the S-SAR M system to achieve a ghost-free image. It must be emphasized that this threshold is inherently system-specific, as it depends on the receiver linearity, noise floor, operating frequency, and front-end architecture of the particular hardware platform employed. Experimental data clearly show that when the isolation is below this threshold, the power of the intermodulation components generated by strong leakage signals through the receiver's third-order nonlinearity exceeds the system noise floor, resulting in identifiable symmetrical artifacts in the image distance direction. When the isolation reached or exceeded 74 dB, the IM3 component was effectively suppressed below the noise floor, completely eliminating the artifact. This is because the leakage signal power is controlled within the linear dynamic range of the receiver. Based on the above core findings, this study not only clarifies the quantitative mapping relationship between isolation and SNR or artifact intensity but, more importantly, clearly defines the key performance boundaries for engineering practice: the critical threshold for eliminating artifacts and the optimal balance range for maintaining peak SNR. Based on this, a spatial parameter design rule is proposed, dominated by elevation-angle control and supplemented by horizontal-spacing coordination at large angles. A direct comparison on the measured data shows that the conventional spacing-only strategy at zero angle cannot cross the artifact-free threshold, whereas the proposed angle-priority rule does. The findings of this study deepen the understanding of how the front-end hardware performance of GB-SAR systems constrains imaging quality from a physical mechanism perspective, providing a physical foundation and actionable technical guidelines for the hardware design of compact Ku-band GB-SAR systems of the studied class, together with a transferable calibration methodology for other platforms.

## 2. Materials and methods

### 2.1. Experimental system and principle

This study was conducted using the Ku-band GB-SAR S-SAR M system developed by the China Institute of Occupational Safety Science and Technology. This system is independently developed by the China Academy of Safety Science and Technology. All experiments were conducted at the internal testing site of the China Academy of Safety Science and Technology in Beijing. Therefore, this study does not require permission from external agencies, and this work does not require any permission. The system is based on synthetic aperture radar principles and differential interferometry (DI). It transmits continuous wave signals in the Ku band via transmit/receive antennas and moves back and forth along a sliding track. By moving a small antenna along a straight track to synthesize a large aperture, two-dimensional high-resolution imaging and high-precision measurements of sub-millimeter-level displacement of slope targets can be achieved. In compact systems, owing to physical size constraints, ensuring adequate spatial isolation between the transmit (Tx) and receive (Rx) antennas is often difficult. Isolation is defined as the signal power attenuation from the transmit port to the receive port, as illustrated in Fig 1, and is typically expressed in decibels as follows.


$$\begin{array}{c} I_{iso}=\text{10}{\text{log}}_{\text{10}}\left(\frac{P_{tx}}{P_{leak}}\right) \end{array}$$ (1)

**Fig 1:**
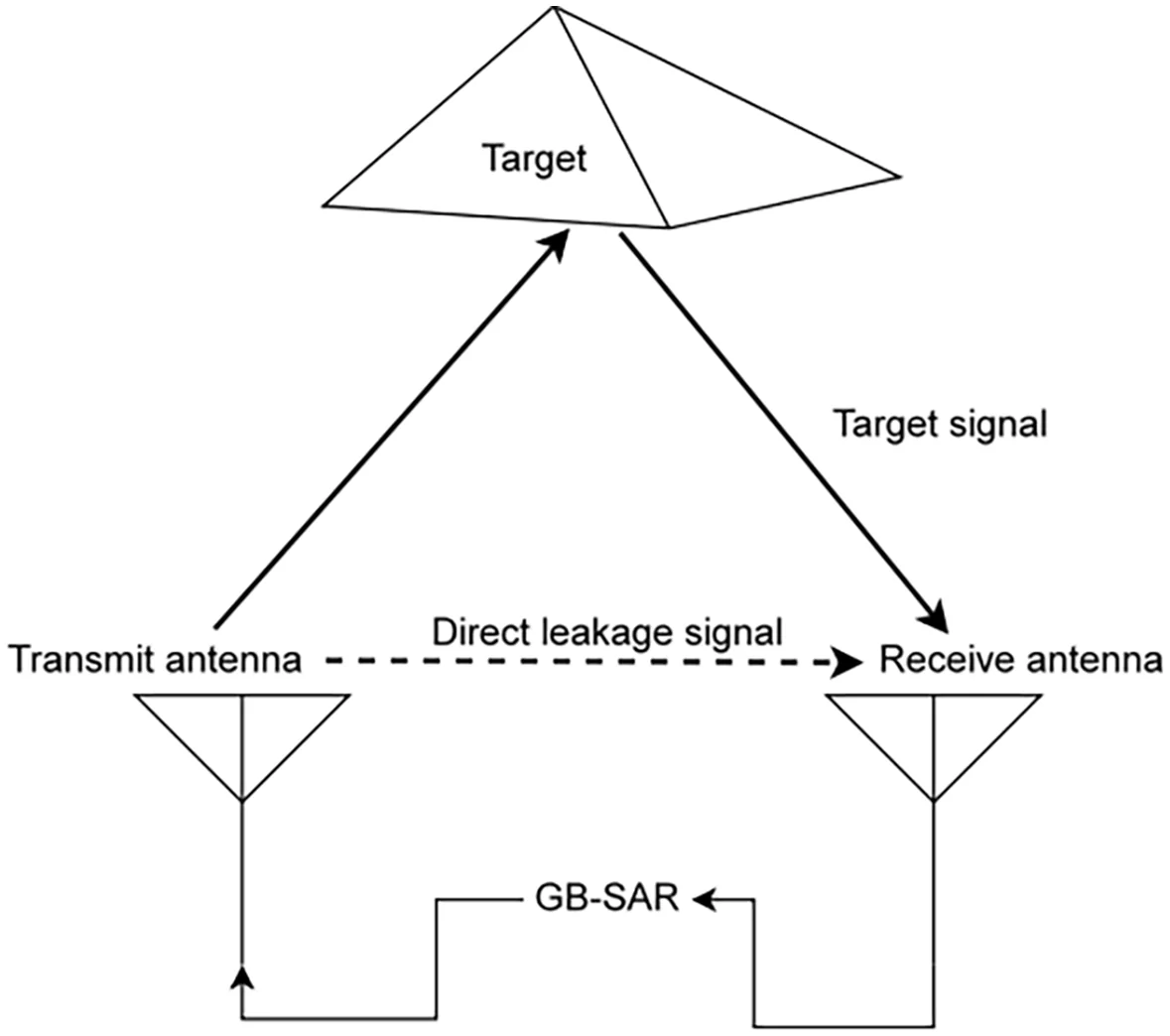
System signal diagram.

Where *P*_*tx*_ represents the transmission power and *P*_*leak*_ represents the power leaked into the receiving channel. Insufficient isolation, corresponding to a low value of *I*_*iso*_, can produce substantial leakage of the transmitted signal when *P*_*leak*_ reaches a large magnitude. This effect constitutes a critical bottleneck for the imaging quality of the GB-SAR system. *I*_*iso*_ is primarily determined by the spatial relative position of the transmitting and receiving antennas, including horizontal spacing d and relative angle(*θ*), together with the shielding structure *C*_*sh*_.

At high frequencies, such as the Ku band, path loss and antenna patterns are the dominant physical mechanisms that affect isolation. The effective isolation between antennas can be modelled as,


$$\begin{array}{c} I_{iso}\left(d,\theta \right)\approx \text{20}{\text{log}}_{\text{10}}\left(\frac{\text{4}\pi }{\lambda }l\left(d,\theta \right)\right)-\left[G_{tx}(\text{0})+G_{rx}\left(\text{0}\right)\right]+\Delta G(\theta )+C_{s\text{h}} \end{array}$$ (2)

Where, *λ* is the radar operating wavelength, and l(d,θ)=d/cosθ is the effective straight-line distance between the phase centres of the transmit and receive antennas. $G_{tx}\left(\text{0}\right)$ and $G_{rx}\left(\text{0}\right)$ are the gains of the transmit and receive antennas at (*θ* = 0°); $\Delta G(\theta )=\left[G_{tx}\left(\theta \right)-G_{\text{tx}}\left(\text{0}\right)\right]+\left[G_{rx}\left(\theta \right)-G_{rx}\left(\text{0}\right)\right]$ is the gain attenuation of the antenna in the *θ* direction relative to the maximum gain. Typically, as *θ* increases, ΔG(θ) becomes negative, and its absolute value increases; $C_{sh}$ is the additional isolation introduced by the shielding structure.

To make formula (2) quantitatively reproducible for the S-SAR M system, all of its terms were calibrated rather than adjusted visually. The co-polar H-plane radiation pattern of the deployed horn was measured in 1° rotation steps, from which the off-boresight gain attenuation Δ G(θ) was obtained for both antennas. The shielding term $C_{\text{sh}}$ is identified at θ = 0°, where Δ G(0)=0: it equals the mean over the four θ = 0° configurations of $\left[I_{\text{iso}}\left(d,\text{0}\right)-\text{20}{\text{log}}_{\text{10}}\left(4\pi d/\lambda \right)\right]$ plus the two boresight gains $G_{\text{tx}}\left(\text{0}\right)+G_{\text{rx}}\left(\text{0}\right)$. The directly measurable intercept $K=C_{\text{sh}}-G_{\text{tx}}\left(\text{0}\right)-G_{\text{rx}}\left(\text{0}\right)$ was determined as 11.3 ± 1.6 dB (mean ± standard deviation). Because rotating the horns also changes the fraction of radiated energy intercepted by the absorbing shield, the quantity identifiable from the 16 measured configurations is the net angular term $G_{\text{a}}\left(\theta \right)=\Delta \;G\left(\theta \right)+\Delta \;C_{\text{sh}}\left(\theta \right)$, which least-squares calibration gives as 22.2 dB, 20.5 dB and 19.2 dB at 30°, 45° and 60°. Using these values, formula (2) yields predictions for all 16 measured isolation data points and achieves an RMSE of 1.8 dB. Removing the single anomalous configuration reduces this RMSE to 1.4 dB. These results demonstrate that the model is physically grounded and independently reproducible.

$I_{iso}$ increases logarithmically with effective distance, but is limited by the gain attenuation ΔG(θ) of the antenna in the azimuth direction. Increasing *θ* significantly increases *l* and this change improves isolation on the one hand, but also exacerbates ΔG(θ) and this effect reduces isolation on the other hand. An increase in *d* on*l*y increases *l*, and its effect is enhanced at a specific *θ*.

When leakage signal $P_{leak}$ enters a high-sensitivity receiving channel, it severely degrades image quality through two main mechanisms, a decrease in signal-to-noise ratio (SNR) and the generation of distance-related artifacts. When leakage signal $P_{leak}$ acts as a strong interference, it significantly raises the equivalent input noise temperature of the receiving system, thereby degrading the system's noise figure. The effective noise power $P_{n,eff}$ of the system can be expressed as,


$$\begin{array}{c} P_{n,eff}\approx P_{n}+P_{leak}\alpha \end{array}$$ (3)

Where, $P_{n}$ is the inherent thermal noise power of the receiver, *α* represents a coefficient associated with the linearity and gain of the receiver, and values of this coefficient stay below one under normal circumstances.

The target echo signal power is denoted by *P*_*s*_. Then, the signal-to-noise ratio (SNR) of the image is,


$$\begin{array}{c} \text{SNR}=\text{10}{\text{log}}_{\text{10}}\left(\frac{P_{s}}{P_{n,eff}}\right)\approx \text{10}{\text{log}}_{\text{10}}\left(\frac{P_{s}}{P_{n}+P_{leak\;}\alpha }\right) \end{array}$$ (4)

When $P_{leak}\alpha >>P_{n}$, values of $I_{iso}$ fall to a sufficiently low level. Under this condition, SNR is mainly dominated by $P_{leak}$ and forms a low-value plateau. As $I_{iso}$ rises while $P_{leak}$ declines, $P_{leak}\alpha$ gradually approaches $P_{n}$ and eventually drops below this threshold. Under this condition, the SNR increases and converges toward the theoretical maximum value $\text{10}{\text{log}}_{\text{10}}\left(P_{s}/P_{n}\right)$.

More seriously, the strong leakage signal $P_{leak}$ and the target echo signal $P_{echo}$ acting together on the nonlinear elements in the receiving chain can produce third-order intermodulation distortion (IM3). The most important IM3 components correspond to frequencies $\text{2}f_{leak}-f_{echo}$ and $\text{2}f_{echo}-f_{leak}$. After pulse compression processing in the range domain, these IM3 components generate false target points. In the imaging results, such points manifest as range-domain artifacts including regular bright lines or ghost targets. The relationship between the power $P_{\text{IM3}}$ of the IM3 products and the input signal power is,


$$\begin{array}{c} P_{\text{IM3}}\propto P_{leak\;}^{\text{2}}P_{echo\;} \end{array}$$ (5)

For a weakly nonlinear receiver, standard third-order distortion theory rewrites formula (5) in terms of measurable receiver parameters. For the intermodulation product $\text{2}f_{\text{leak}}-f_{\text{echo}}$, the leakage tone appears twice and the echo tone appears once. The input-referred IM3 power, expressed in dBm, is


$$\begin{array}{c} P_{\text{IM3}}=\text{2}P_{\text{leak}}+P_{\text{echo}}-\text{2IIP3} \end{array}$$ (6)

Where IIP3 is the third-order input intercept point. Linear operation additionally requires the leakage to remain below the receiver 1-dB compression point $P_{\text{1dB}}$, related to IIP3 by $P_{\text{1dB}}\approx \text{ IIP3}-\text{9}.\text{6}$ dB, so that gain compression cannot precede intermodulation. Equating the IM3 power to the receiver noise floor $P_{\text{n}}$ then yields the analytical critical isolation threshold


$$\begin{array}{c} I_{\text{iso},\text{th}}=P_{\text{tx}}-\frac{P_{\text{n}}+\text{2IIP3}-P_{\text{echo}}}{\text{2}} \end{array}$$ (7)

Formula (7) identifies the four receiver-level parameters that set the critical threshold, namely transmit power, echo level, noise floor and IIP3. This set of parameters is equivalent to the 1-dB compression point. Therefore, the formula explains the physical origin of the threshold rather than relying on empirical assertion. For the S-SAR M system where $P_{\text{tx}}$ equals 30 dBm, the measured 74 dB threshold corresponds to a maximum tolerable leakage of −44 dBm at the receive port. Formula (7) also defines the generalized extrapolation route. When working with any other GB-SAR platform, substituting the four corresponding parameters yields a platform-specific threshold and avoids direct reuse of the 74 dB numerical value.

The visibility of artifacts depends on the ratio of $P_{\text{IM3}}$ to the system noise baseline $P_{n}$. There is a critical isolation threshold $I_{iso\;}^{th\;}$, when $I_{iso}\geq I_{iso\;}^{th\;}$, satisfying,


$$\begin{array}{c} P_{\text{IM3}}<P_{n} \end{array}$$ (8)

At this point, the IM3 product is drowned out by noise, and the distance-related artifacts are invisible in the image. Conversely, when $I_{iso}<I_{iso\;}^{th\;}$, $P_{\text{IM3}}\geq P_{n}$, the artifacts inevitably appear, and their intensity increases exponentially as $I_{iso}$ decreases ($P_{leak}$ increases).

Increasing *θ* or *d* can enhance $I_{iso}$, thereby suppressing leakage, improving SNR, and eliminating artifacts. However, excessive values of *θ* and d can lead to new issues. Physical separation between the transmit and receive antennas leads to larger values of *d*, and angular deflection produces larger values of *θ*. These two effects jointly make their respective beam centers deviate from the ideal radar axis centerline. The offset of the beam center violates the phase center consistency assumption relied upon for ideal GB‑SAR imaging. This spatial offset introduces spatial phase errors Δϕ(x,r) during synthetic aperture processing, where x is the antenna position and r is the target distance. Phase errors reduce the effective gain $G_{syn}$ of the synthetic aperture,


$$\begin{array}{c} G_{syn}\propto {\left|\frac{\text{1}}{N}\sum_{k=\text{1}}^{N}\;e^{j\Delta \phi \left(x_{k},r\right)}\right|}^{\text{2}} \end{array}$$ (9)

Where *N* is the number of synthetic aperture sampling points. This ultimately leads to a decrease in the target echo signal power $P_{s}$, causing the SNR to drop in the high isolation region. The key parameters of the S-SAR M system are presented in Table 1.

**Table 1:** Key specifications of the S-SAR M system.

Parameters	Numerical
Centre frequency	16.4GHz
Signal bandwidth	1000MHz
3dB beam width	120°(H)×30°(V)
Monitoring distance	5000 m
Antenna type	Horn antenna
Distance-to-resolution	0.15m
Azimuth resolution	4mrad
Synthetic aperture length	2m
Accuracy	0.1mm

For the S-SAR M parameters, the wavelength *λ* equals 18.3 mm, the synthetic aperture length *L* equals 2 m, and the corner-reflector test distance *r* equals 24 m. This gain loss can be quantified under such parameter conditions. The lateral phase-center offset is ϵ=d/sinθ and the maximum differential phase across the aperture before focusing is $\Delta \phi_{\text{max}}=(2\pi /\lambda )(\epsilon L/\text{2}r)$, which ranges from 1.3 rad at (30°, 175 mm) to 2.8 rad at (60°, 225 mm) across the tested configurations. Standard SAR focusing removes the linear phase ramp, leaving a residual root-mean-square phase error $\sigma_{\varphi }$ of 0.6–0.9 rad; by the Ruze relation $G_{\text{syn}}/G_{\text{0}}\approx \text{exp}\left({-\sigma }_{\varphi }^{\text{2}}\right)$ this corresponds to a synthetic-aperture gain loss of 1.6–3.5 dB, quantitatively consistent with the measured 2.5 dB SNR decline from the 41.2 dB peak in the high-isolation region.

### 2.2. Experimental device and parameter regulation

To quantify the impact of antenna spatial parameters, namely angle(*θ*) and horizontal spacing d, on the transmit-receive isolation, we designed and constructed a high-precision adjustable antenna device based on the S-SAR M system platform, as shown in Fig 2. This device supports continuous adjustment of the horizontal spacing d over a mechanical travel of 25–250 mm and independent adjustment of the relative angle θ between the transmit and receive antennas from 0° to 60°.

**Fig 2:**
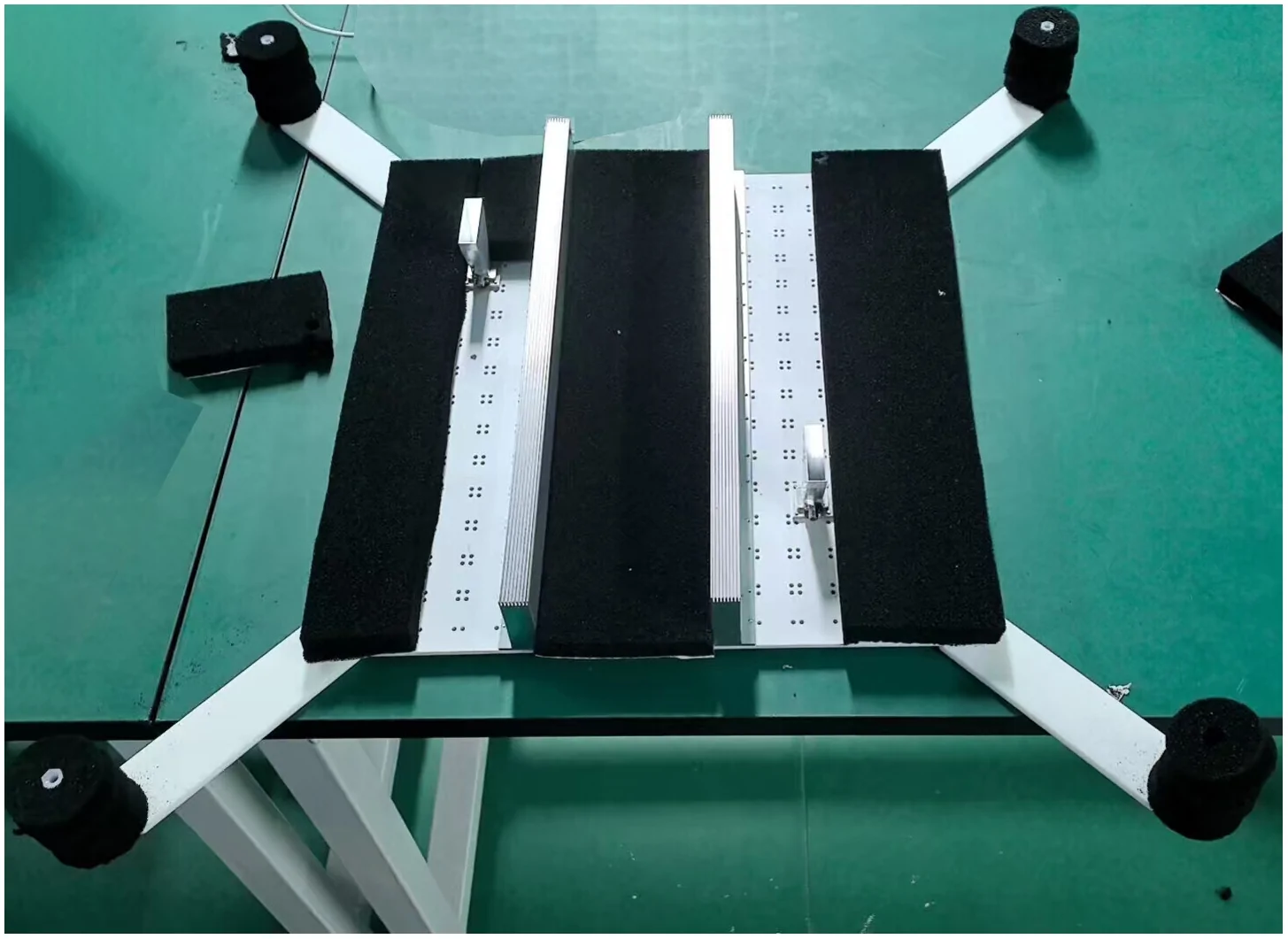
Antenna adjustment mechanism.

An orthogonal experimental design was adopted in this work. The radar operating conditions were set with a center frequency at 16.4 GHz and a bandwidth at 1 GHz, and the transmission power remained fixed. The horizontal spacing d and angle θ combinations of the antennas were systematically adjusted, and a vector network analyzer was used to precisely measure the effective straight-line distance between the antennas and the isolation between the transmit and receive ports for each configuration of the antenna. The isolation measurements were repeated five times for each set of parameter configurations, and the arithmetic mean was considered the final result to reduce the random errors. Preliminary scans across the full mechanical travel range that extends from 25 mm to 250 mm revealed that isolation stays below 53 dB when *d* remains at or below 150 mm. Isolation values fall at or below 50 dB for *d* at or below 100 mm. Under these circumstances, direct-coupling leakage dominates the receiver response, and distinctions among different configurations cannot be identified. This sub-range was consequently excluded from the formal experiment, whose effective domain is d = 150–225 mm at four levels and θ = 0°, 30°, 45°, 60°, as listed in Table 2; the 25–250 mm mechanical travel and the 150–225 mm effective test domain are therefore distinct by design, not contradictory. To verify robustness, the dispersion of the five repeats was examined at every configuration, and one-way analysis of variance (ANOVA) across angle groups together with Welch's t-tests between isolation zones was performed. Experimental simulations were conducted using the same parameter configuration matrix, and the simulation results are presented in Fig 3.

**Table 2:** Antenna spatial configuration versus measured isolation.

Serial number	Angle/°	Antenna horizontal spacing/mm	Antenna straight line distance/mm	Isolation/dB
1	0	150	150	53.3
2	30	150	173.2	73.3
3	45	150	212.1	73.1
4	60	150	300	74.6
5	0	175	175	53.7
6	30	175	202.1	77.2
7	45	175	247.5	78.1
8	60	175	350	82.9
9	0	200	200	52.7
10	30	200	230.9	78.7
11	45	200	282.8	78.4
12	60	200	400	78.2
13	0	225	225	53.7
14	30	225	259.8	77.8
15	45	225	318.2	77.7
16	60	225	450	78.5

**Fig 3:**
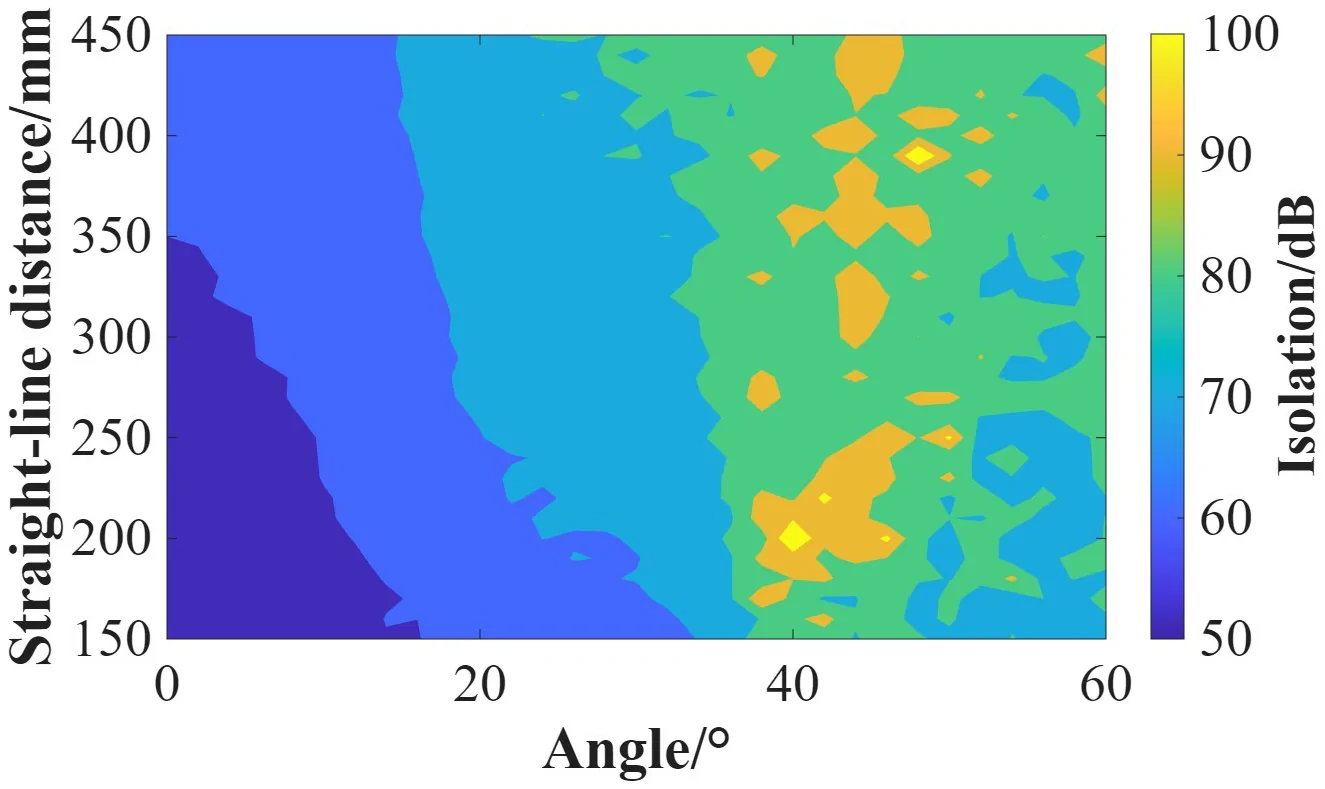
Simulated isolation versus antenna spatial parameters.

### 2.3. Signal-to-noise ratio (SNR) assessment

For the selected antenna spatial parameter configuration, echo data were acquired from a standard corner reflector with an RCS of 20 dBsm placed at a fixed position. These data were imaged under a typical test scenario, as illustrated in Fig 4, to quantitatively evaluate the imaging quality.

**Fig 4:**
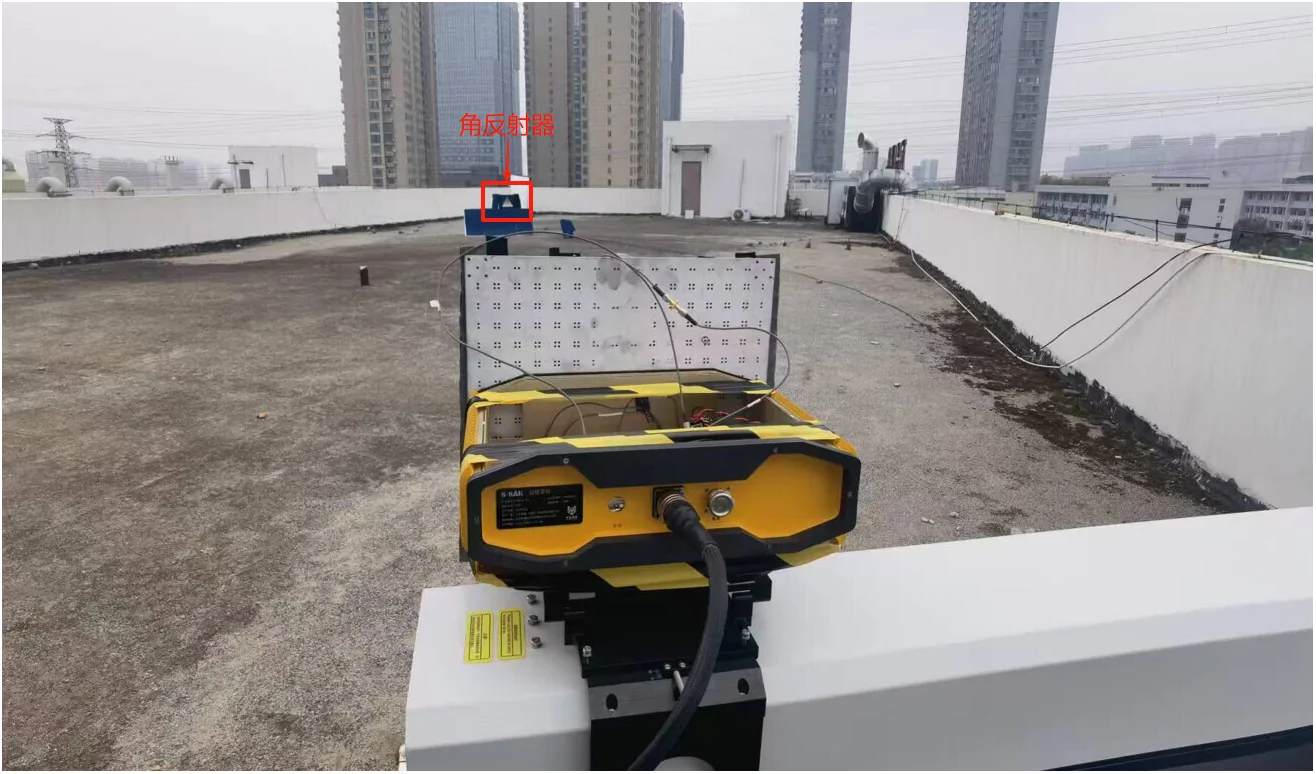
Test scenario.

Each set of parameter configurations was repeated five times in the imaging experiment, and the average SNR was calculated as the final result. SNR was computed as the ratio, in dB, of the peak echo power of the corner reflector to the mean background power averaged over an equal-area artifact-free annular window. An example of the imaging results for the corner reflector is shown in Fig 5.

**Fig 5:**
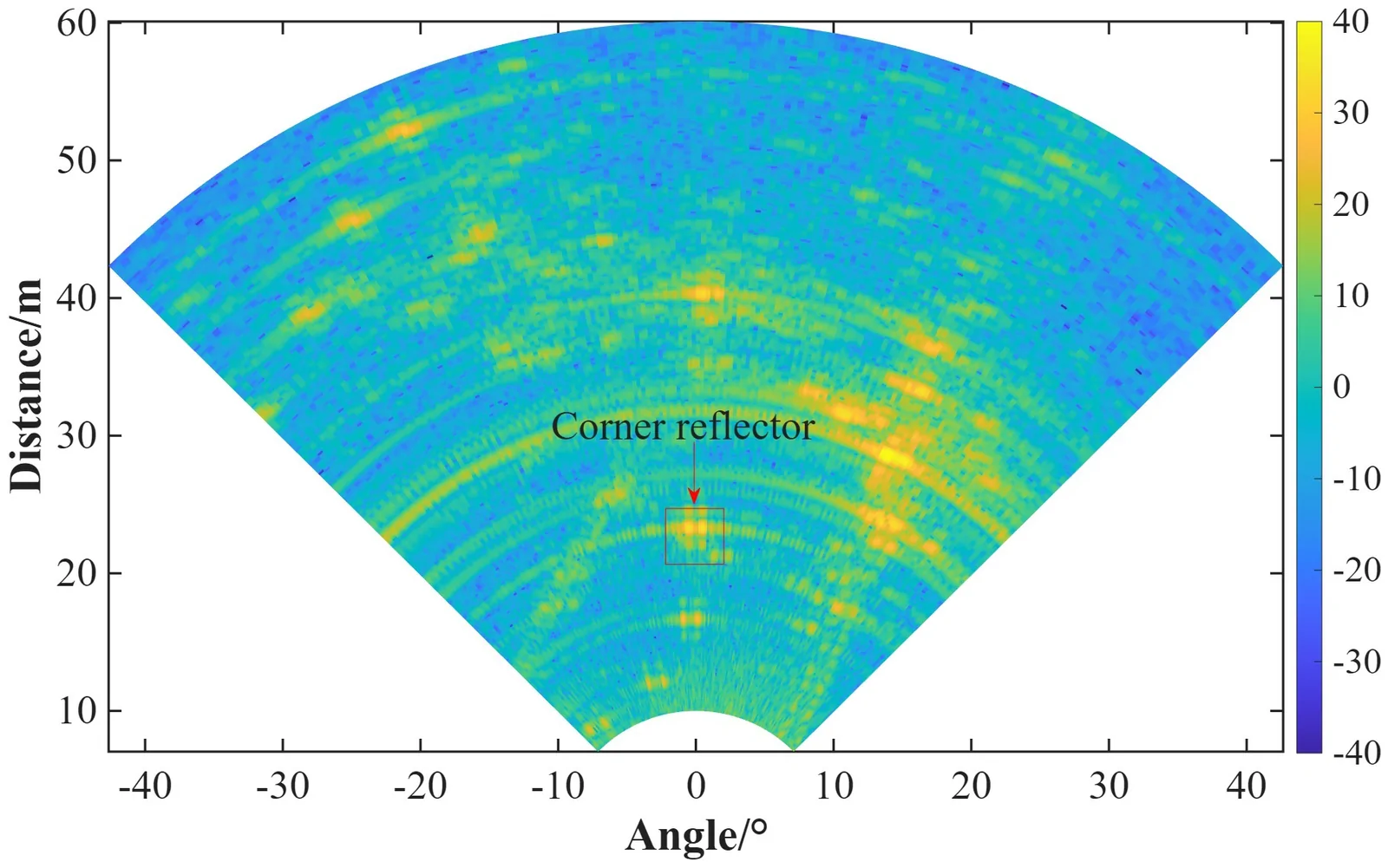
Radar imagery results.

### 2.4. Distance artifact assessment

The radar imaging distance range was set to span from 0 m to 500 m. The imaging map was visually inspected to identify distance-related artifacts induced by intermodulation distortion. Such artifacts appear as vertical lines or localized bright features that cross the image. Artifact severity is defined in three categories. Strong artifacts correspond to obvious vertical interference lines that run across the whole imaging area. Weak artifacts represent local interference features that do not cover the entire image. No artifacts indicate clean images without obvious interference structures. Typical artifact-free and strong/weak artifact comparison imaging diagrams are shown in Fig 6–8. To complement the visual classification with a quantitative, operator-independent measure, an artifact-to-background energy ratio (AER) was defined as


$$\begin{array}{c} \text{AER}=\text{10}{\text{log}}_{\text{10}}\left(P_{\text{art}}/P_{\text{bg}}\right) \end{array}$$ (10)

**Fig 6:**
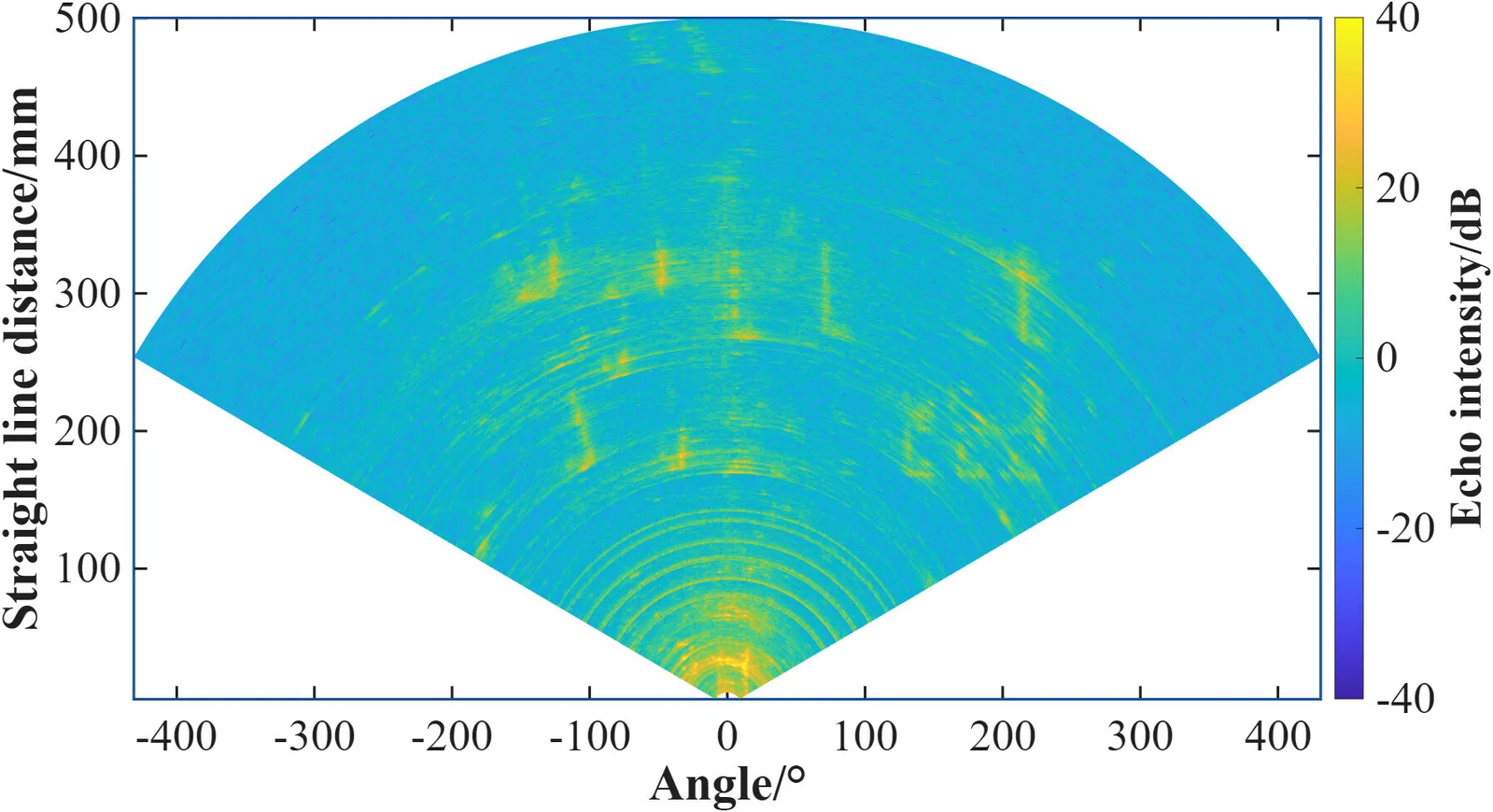
Ghost-artifact-free imaging example.

**Fig 7:**
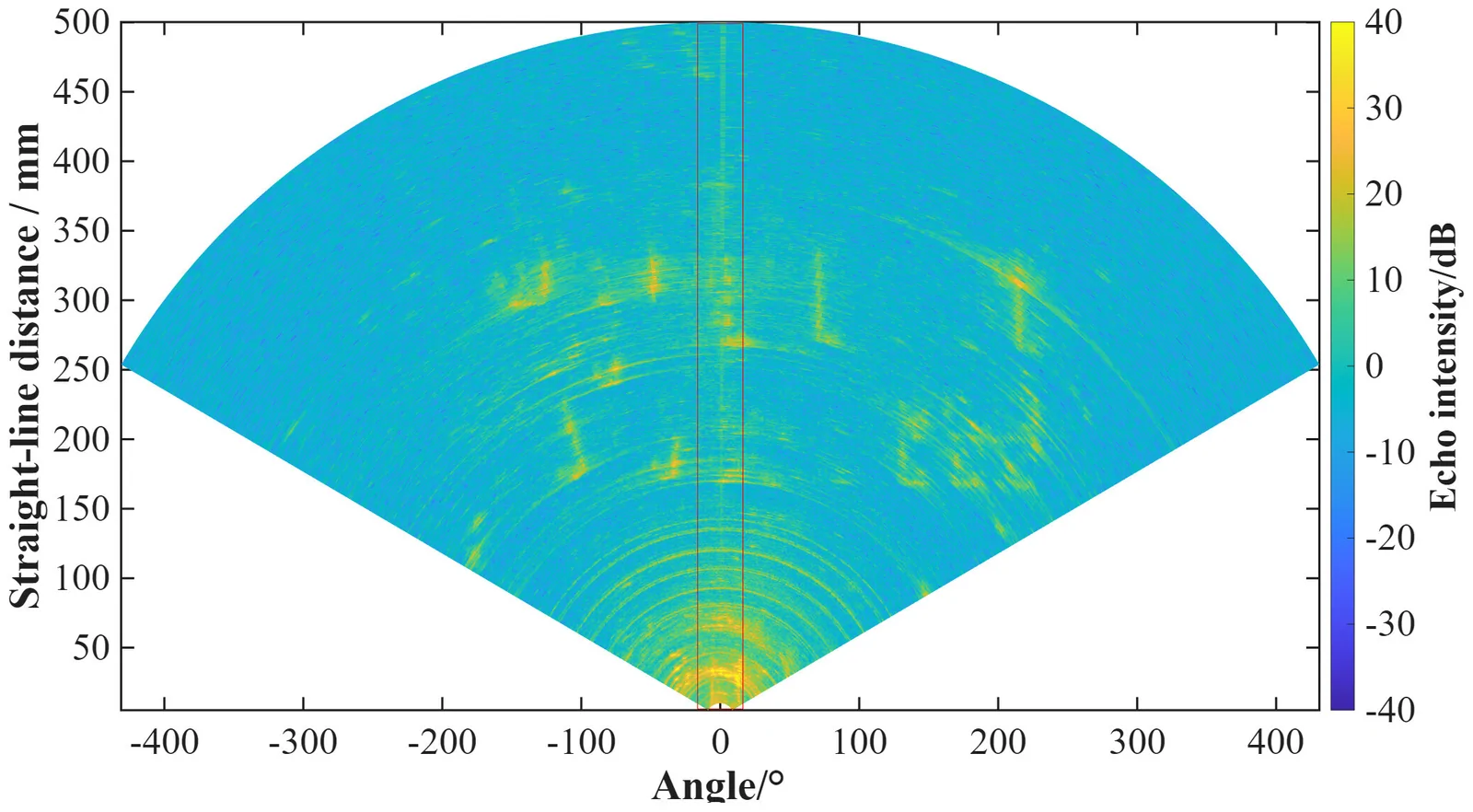
Strong ghost artifacts imaging example.

**Fig 8:**
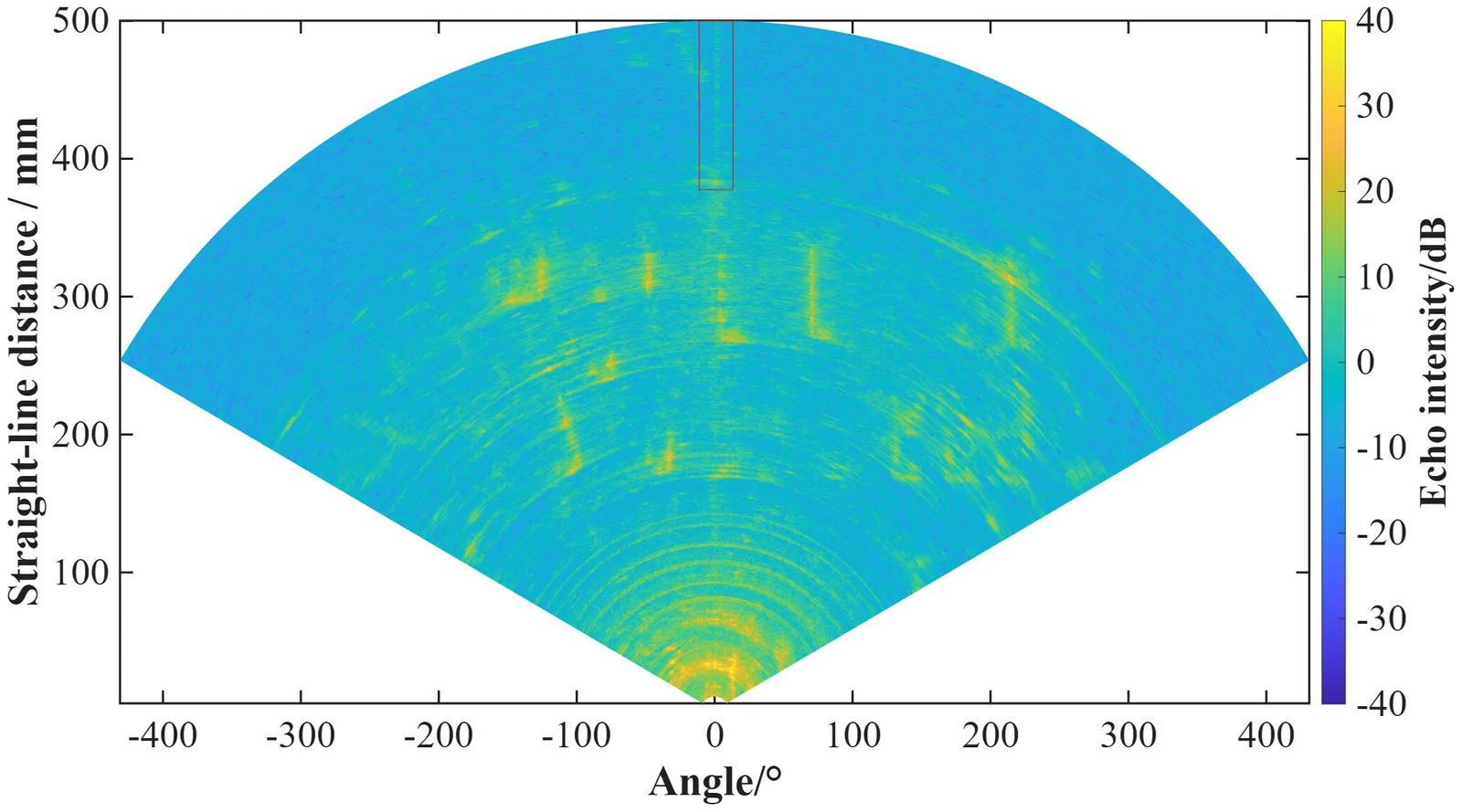
Weak ghost artifacts imaging example.

Where $P_{\text{art}}$ is the mean pixel power over the range-direction artifact region and $P_{\text{bg}}$ is the mean power of an equal-area artifact-free background window. From the joint distribution of AER and the visual labels over all 16 configurations, no artifacts correspond to AER < 3 dB, weak artifacts to 3–6 dB, and strong artifacts to AER > 6 dB. Two operators independently carried out visual labeling and AER calculation for every image. A high level of agreement exists between these two operators, and Cohen's *κ* achieves a value of 0.87. The AER values presented in the following content are the averaged results obtained from the two operators.

## 3 Results

### 3.1. Quantitative influence of spatial parameters on isolation degree

To explore the influence of the antenna spatial parameters on isolation, this study measured the isolation values under different angle and horizontal spacing combinations. Table 2 presents specific data.

The orthogonal experimental data presented in Table 2 indicate that the antenna angle θ and straight-line distance d exert a significant nonlinear coupling effect on the isolation degree, as illustrated in Fig 9.

**Fig 9:**
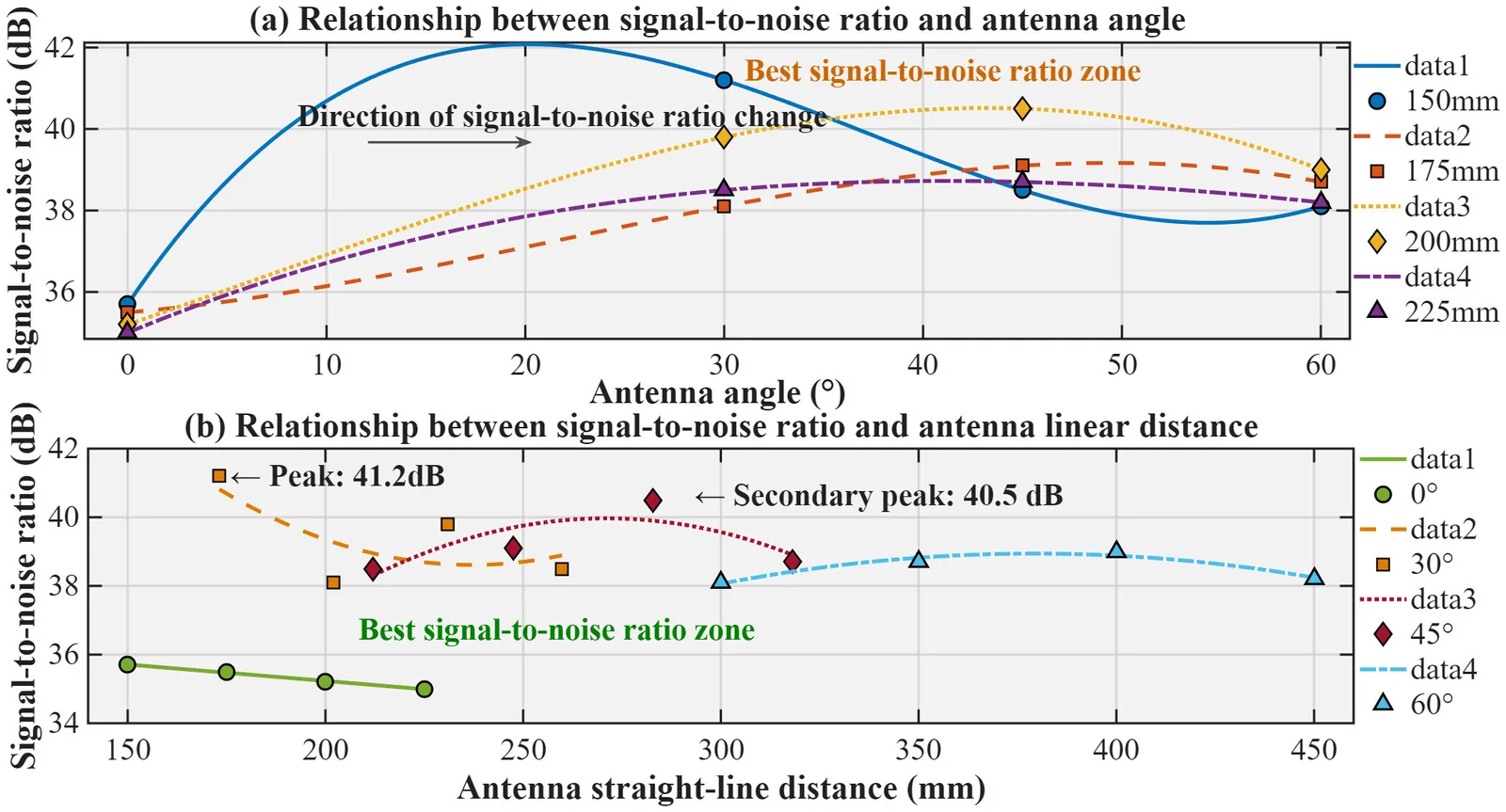
Isolation variation curves vs. antenna azimuth angle and linear distance.

The angle is the primary factor influencing the isolation performance. When the angle increases from 0° to 60°, the isolation performance improves by an average of 56.3%. As an example, the isolation value rises from 53.3 to 74.6 dB under a fixed distance d of 150 mm. This phenomenon is highly consistent with the theoretical isolation performance model, and the calibrated formula (2) reproduces these measurements within an RMSE of 1.8 dB.

When θ ≥ 30°, increasing the distance resulted in a synergistic gain in isolation performance. For every 100 mm increase in distance, the isolation performance improved by an average of 4.2 dB. Regions with high isolation performance above 74 dB are concentrated in the parameter space where θ > 45° and d > 300 mm. As an example, the parameter combination of 60° and 350 mm yields an isolation value of 82.9 dB.

At an angle of 0°, the effect of distance on isolation was negligible, with fluctuations below 1 dB. This observation further confirms that angle control serves as the core method for breaking through the isolation bottleneck in compact Ku-band GB-SAR designs.

A direct, data-based comparison with the conventional single-parameter strategy clarifies the advantage of the proposed angle-priority rule. With the angle fixed at 0°, varying spacing alone changes isolation only from 52.7 to 53.7 dB and never approaches the 74 dB artifact-free threshold; in contrast, the proposed angle-priority adjustment reaches 74.6–82.9 dB at θ = 60°, a measured 21–29 dB improvement over the best spacing-only configuration. Obtained on the same hardware under the same test protocol, this is the empirical evidence that the dynamic spatial strategy outperforms a static, single-parameter design.

The single non-monotonic point at 60° was examined quantitatively using electromagnetic first principles. This point corresponds to an isolation level of 78.2 dB at 200 mm and 82.9 dB at 175 mm. At 16.4 GHz, the wavelength *λ* equals 18.3 mm. The first Fresnel-zone radius for a distance *l* ranging from 300 mm to 450 mm falls between 52 mm and 64 mm, comparable to the dimensions of the absorbing shielding panels and the mounting fixture. A 25 mm spacing increment changes the round-trip coupling path by 50 mm, which equals approximately 2.7 wavelengths. This change rotates the phase of the fixture-reflected coupling component by approximately 17 rad and shifts this component repeatedly between constructive and destructive superposition with the direct path. The Fraunhofer distance of the horn aperture ranging from 35 mm to 40 mm is only 134 mm to 175 mm, so the two horns lie within each other's far field while the structural coupling paths remain in the radiating near field. This analysis shows that the observed local ripple ranging from −2 dB to +5 dB is the expected consequence of near-field multipath between the horns and the fixture rather than random measurement error. The dip was reproduced in all five repeats under this configuration. Therefore, this phenomenon does not alter the statistically dominant angle trend presented below.

### 3.2. Spatial response characteristics of signal-to-noise ratio

The standard corner reflector was imaged under different spatial parameter configurations, and its signal-to-noise ratio (SNR) was determined. The results are shown in Table 3.

**Table 3:** Signal-to-noise ratio test data.

Serial number	Angle/°	Antenna horizontal spacing/mm	Antenna straight line distance/mm	SNR/dB
1	0	150	150	35.7
2	30	150	173.2	41.2
3	45	150	212.1	38.5
4	60	150	300	38.1
5	0	175	175	35.5
6	30	175	202.1	38.1
7	45	175	247.5	39.1
8	60	175	350	38.7
9	0	200	200	35.2
10	30	200	230.9	39.8
11	45	200	282.8	40.5
12	60	200	400	39.0
13	0	225	225	35.0
14	30	225	259.8	38.5
15	45	225	318.2	38.7
16	60	225	450	38.2

Combined with the data in Table 3, the SNR exhibits a distinctive non-monotonic bell shaped response characteristic as spatial parameters θ and D vary, as shown in Fig 10.

**Fig 10:**
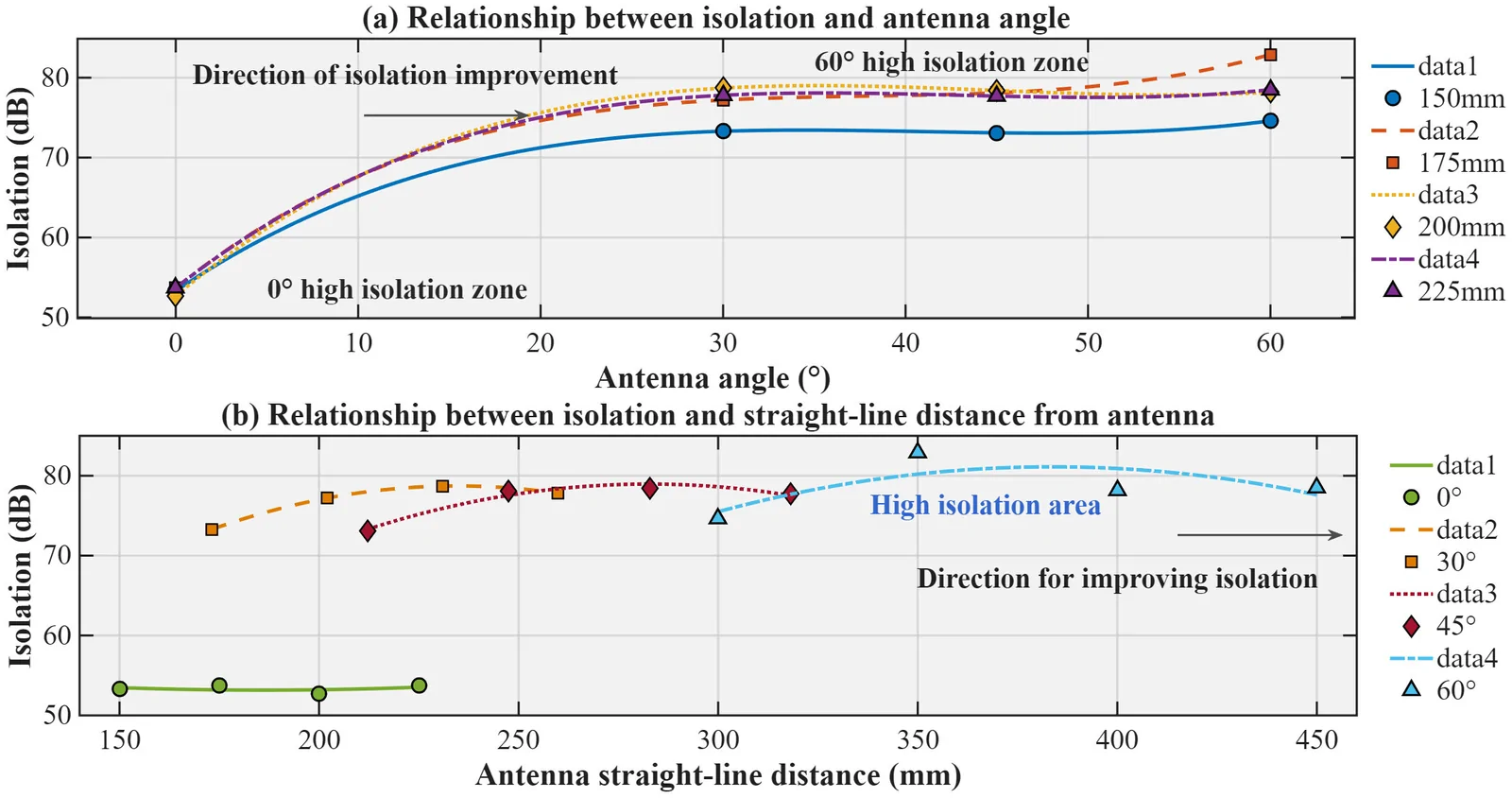
SNR variation curves vs. antenna azimuth angle and linear distance.

In the angular dimension, the SNR reaches a peak of 41.2 dB at θ = 30°. This value represents an increase of approximately 15.8% compared with the result obtained at 0°, and the SNR subsequently decreases by 7.6% as the angle rises to 60°. In the distance dimension, an optimal range exists where d varies from 170 mm to 280 mm. Within this range, the SNR generally exceeds 39.5 dB.

This non-monotonic change stems from the double-edged sword effect of spatial parameter adjustments. Distances below 150 mm introduce strong direct coupling leakage signals into the receiving channel. These leakage signals not only directly increase the equivalent noise figure of the system but also generate significant third-order intermodulation distortion (IM3) with the target echo in the receiver's nonlinear components, leading to a sharp deterioration in the SNR.

When d exceeds 350 mm or θ exceeds 45°, isolation is significantly enhanced, which suppresses leakage and IM3. Nevertheless, the physical separation between the transmit and receive antennas causes the beam center to shift further away from the radar centerline. This offset disrupts the phase center consistency required for ideal synthetic aperture imaging, introducing additional spatial phase errors and reducing the effective gain of the synthetic aperture, thereby weakening the target signal strength and ultimately causing a decline in the SNR. This phenomenon is consistent with the sensitivity of the synthetic aperture gain to phase errors in theoretical models.

The significance and robustness of these trends were tested statistically. A one-way ANOVA across the four angle groups, with four configurations within each group, shows that angle exerts a highly significant effect on both isolation and SNR. The corresponding statistical metrics are F(3,12) = 97.9 with p less than 0.001 for isolation and F(3,12) = 18.5 with p less than 0.001 for SNR. The mean SNR in the low-isolation zone reaches 35.35 ± 0.31 dB for a sample size n of 4, and this value is significantly lower than the mean SNR in the optimal balance zone, which equals 38.89 ± 0.82 dB for a sample size n of 9. The Welch's t-test yields t = 11.3, df = 11 and p less than 0.001. This result confirms that the threshold and optimal-zone conclusions are not attributable to random measurement error. The 2.5 dB SNR decline observed within the high-isolation region agrees quantitatively with the synthetic-aperture gain loss spanning 1.6 dB to 3.5 dB, instead of originating from random fluctuation.

### 3.3. Nonlinear coupling mechanism between isolation and signal-to-noise ratio

Based on the data in Tables 2 and 3, the coupling relationship between the isolation and SNR is shown in Fig 11. There is a three-stage nonlinear coupling relationship between the two, and its change law can be well fitted using a quadratic function model (R^2^ = 0.92). This quadratic envelope is the reduced empirical form of a mechanistic composite model rather than an arbitrary polynomial fit: in the low-isolation regime formula (4) makes SNR rise monotonically as leakage is suppressed, whereas in the high-isolation regime formula (9) imposes a synthetic-aperture gain loss that grows quadratically with beam offset, and their superposition gives


$$\begin{array}{c} \text{SNR}(I)\approx {\text{SNR}}_{\text{max}}-\text{10}{\text{log}}_{\text{10}}\left[{\text{1}+\text{10}}^{\left(I_{\text{b}}-I\right)/\text{10}}\right]-\kappa (I-I_{\text{0}}{)}_{+}^{\text{2}} \end{array}$$ (10)

**Fig 11:**
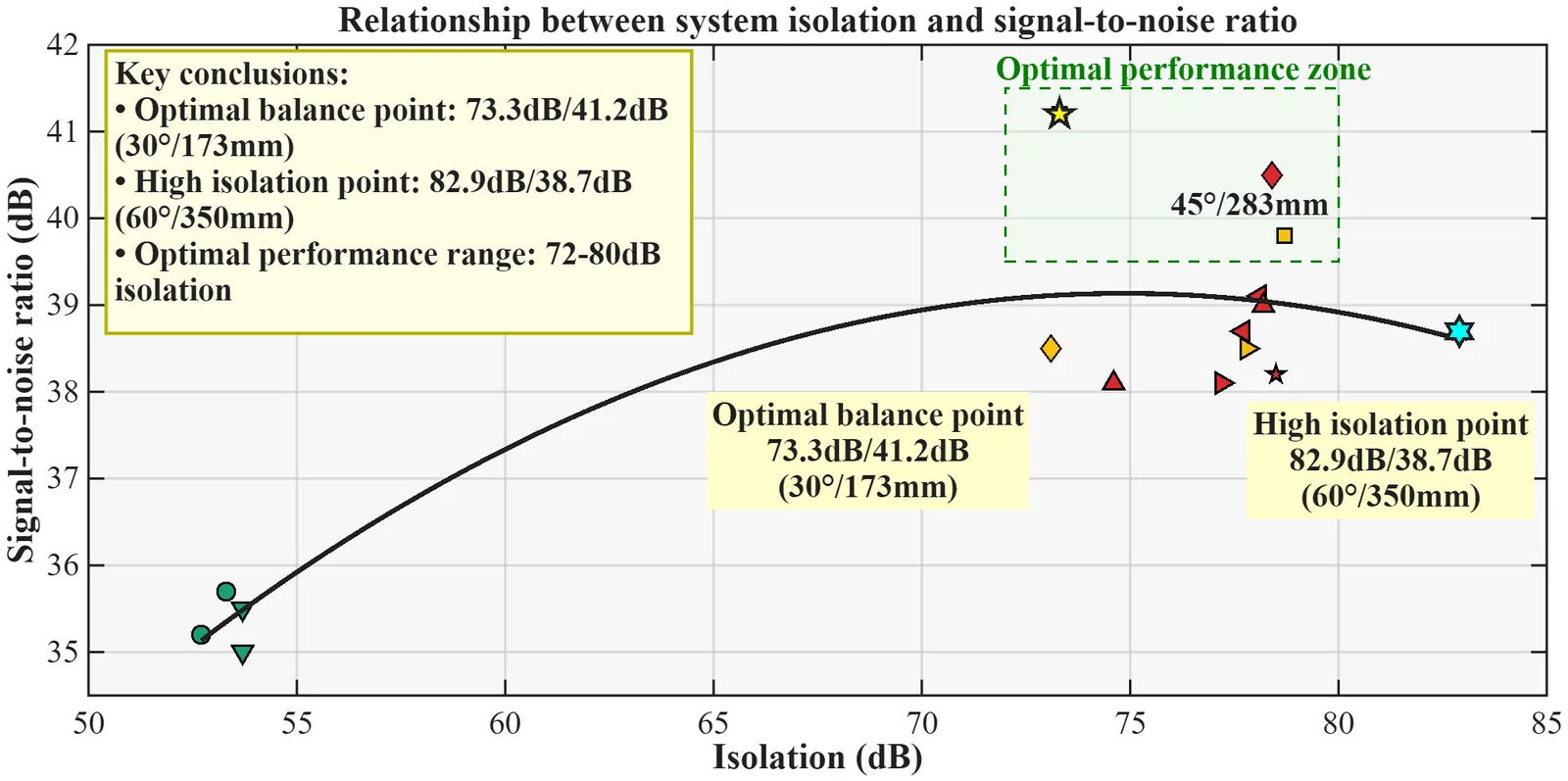
Coupling relationship between isolation and SNR.

Where the first penalty is the leakage-noise floor with breakpoint $I_{\text{b}}$, the second penalty $\kappa (I-I_{\text{0}}{)}_{+}^{\text{2}}$ is the aperture-loss term with $(x{)}_{+}=\text{max}\left(x,\text{0}\right)$, and κ is a platform-specific constant. The crossover of these two analytic mechanisms generates the observed peak, providing the physical derivation that underlies the quadratic fit.

Low isolation zone with isolation below 60 dB: The SNR is dominated by leakage signals and remains at a low level of 35.5 ± 0.5 dB. At this point, the leakage power Pr is significantly higher than the thermal noise Pn, resulting in a sharp deterioration in the effective signal-to-noise ratio (SNR). The experimental results validate the accuracy of the theoretical model for the leakage-noise-dominated mechanism.

Optimal balance zone with isolation ranging from 74 dB to 80 dB: The SNR reaches a peak plateau that ranges from 39.5 dB to 41.5 dB. Within this zone, improved isolation effectively suppresses third-order intermodulation distortion (IM3), reducing it below the thermal noise level and preventing it from dominating system noise. Meanwhile, the antenna spatial configuration has not yet introduced significant synthetic aperture gain loss caused by beam offset. Therefore, the system maintains an optimal balance between suppressing leakage noise and preserving high signal gain.

High-isolation region with isolation above 80 dB: The SNR begins to show a declining trend. As an example, the SNR equals 38.7 dB under an isolation level of 82.9 dB. This is primarily due to the extreme spatial configuration required to achieve such high isolation, which induces a significant beam-center offset. Although isolation reaches its maximum and IM3 distortion is fully suppressed, synthetic aperture gain loss acts as the primary factor limiting the SNR. Therefore, this region is not the optimal operating region for SNR.

### 3.4. Isolation threshold effect of imaging artifacts

The evaluation results of the imaging artifacts at different isolation degrees are listed in Table 4. The artifact level directly reflects the degree of IM3 distortion in the image quality.

**Table 4:** Ghost artifacts evaluation data.

Serial number	Angle/°	Antenna horizontal spacing/mm	Antenna straight-line distance/mm	Artifact level
1	0	150	150	Strong
2	30	150	173.2	Weak
3	45	150	212.1	Weak
4	60	150	300	None
5	0	175	175	Strong
6	30	175	202.1	None
7	45	175	247.5	None
8	60	175	350	None
9	0	200	200	Strong
10	30	200	230.9	Weak
11	45	200	282.8	None
12	60	200	400	None
13	0	225	225	Strong
14	30	225	259.8	Weak
15	45	225	318.2	None
16	60	225	450	None

The experimental data listed in Table 4 reveal that the isolation degree follows a distinct step-like threshold control law for imaging artifacts, as illustrated in Fig 12.

**Fig 12:**
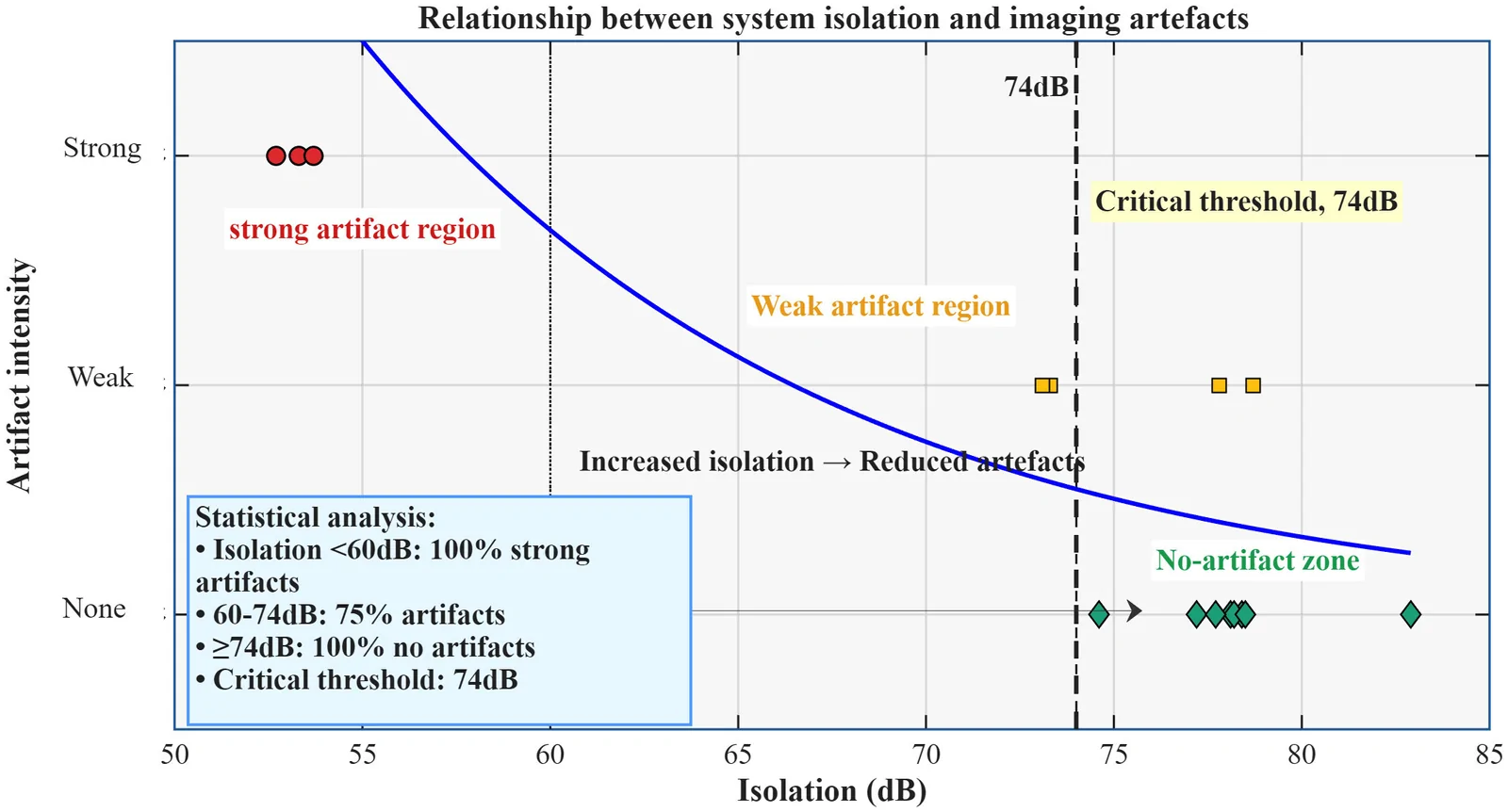
Occurrence of imaging ghost artifacts versus isolation.

Strong artifact region with isolation below 60 dB: All tests exhibited strong artifacts throughout the imaging region. The physical root cause lies in the third-order intermodulation (IM3) distortion components generated by strong leakage signals and target echoes in the nonlinear section of the receiver. These IM3 components manifest as false targets with specific patterns after range compression; that is, artifacts. The relationship between the artifact intensity and isolation degree conforms to the theoretical model, exhibiting exponential growth as *I*_*iso*_ decreases.

Transition Zone with isolation degree ranging from 60 dB to 74 dB: The artifact intensity decreases exponentially with increasing isolation degrees. The fitting results indicate that


$$\begin{array}{c} I_{artifact}\propto e^{-\text{0}.\text{08}(Iso-\text{55})} \end{array}$$ (11)

Within this range, approximately 75% of the test configurations exhibited only localized weak artifacts or no artifacts. Statistical analysis shows that for every 1 dB increase in isolation, the risk of significant artifacts decreases by approximately 15%, indicating that the IM3 distortion is partially suppressed but not completely eliminated.

The quantitative AER metric independently confirms this three-level classification: the mean AER is 7.6 ± 1.0 dB for the four strong-artifact configurations, 4.4 ± 0.9 dB for the four weak-artifact configurations, and 1.3 ± 0.8 dB for the eight artifact-free configurations. Every configuration with isolation at or above 74 dB has an AER below 3 dB, so the independently computed AER boundary coincides exactly with the 74 dB isolation threshold, providing operator-independent quantitative support for the threshold conclusion.

No-artifact zone with isolation at or above 74 dB: All configurations that satisfy this isolation condition deliver pure imaging results and fully eliminate distance-related artifacts induced by intermodulation distortion. The physical mechanism is that the leakage power *P*_*r*_ is suppressed below the thermal noise *P*_*n*_, thereby suppressing the IM3 distortion. According to nonlinear distortion theory, the intensity of the resulting intermodulation products falls below the noise floor and becomes invisible in imaging results if the leakage signal power stays under a threshold determined by the linearity and noise floor of the receiver.

Experimental data confirm that 74 dB is the critical isolation threshold for achieving artifact-free imaging in S-SAR M systems. This conclusion combines physical clarity and engineering guidance.

## 4. Discussion

Through systematic experiments, this study quantifies the mechanism of the impact of transceiver isolation on GB-SAR imaging quality and reveals the key role of spatial parameter regulation in improving the isolation. Compared with previous studies, this work not only defines the quantitative relationship between isolation and imaging quality for a specific Ku-band compact GB-SAR platform but also identifies the nonlinear coupling characteristics and threshold effect, which provides more refined theoretical guidance for the design of GB-SAR systems. While the general principle that antenna angle and spacing influence isolation is recognized in antenna theory, this study contributes specific quantitative data and coupling laws for compact Ku-band GB-SAR configurations, where the trade-off between isolation improvement and synthetic aperture gain loss is particularly acute due to the constrained physical dimensions. The incremental value lies in transforming qualitative engineering intuition into experimentally validated quantitative boundaries and an optimal operating zone, which was previously unavailable in the open literature for this class of systems. To lift the result from a single-platform calibration toward a transferable framework, define the normalized isolation margin $M=I_{\text{iso}}-I_{\text{iso},\text{th}}$, where $I_{\text{iso},\text{th}}$ follows analytically from formula (7): artifact-free operation corresponds to M ≥ 0 and the optimal balance zone to 0 ≤ M ≤ 6 dB (the S-SAR M interval 74–80 dB is one realization). For a new platform, designers (i) measure $P_{\text{tx}}$, $P_{\text{echo}}$, the noise floor and IIP3; (ii) compute $I_{\text{iso},\text{th}}$ from formula (7); (iii) apply angle-priority spatial adjustment until M ≥ 0; and (iv) retain a margin no larger than about 6 dB to avoid synthetic-aperture gain loss. This procedure transfers the methodology without transplanting the numerical value 74 dB.

The experimental results show that the antenna angle is the dominant factor in improving the isolation. This study further quantified the amplitude of the isolation improvement caused by the angle change. Simultaneously, it was found that the spacing can produce synergy at a large angle, which has been less systematically described in the GB-SAR literature. This suggests that in the design of a compact GB-SAR system, angle control should be prioritized, and appropriate spacing optimization should be supplemented when space allows, rather than simply pursuing the maximum spacing. It is worth noting that an anomalous data point was observed where the isolation at 60 degrees and 200 mm spacing was slightly lower than that at 60 degrees and 175 mm spacing. A quantitative near-field analysis in Section 3.1 traces this non-monotonic behavior to multipath effects between the horns and the mounting fixture within the first Fresnel zone. The corresponding radius ranges from 52 mm to 64 mm when l varies from 300 mm to 450 mm. A 25 mm spacing increment rotates the reflected-coupling phase by approximately 17 rad, which equals 2.7 wavelengths. This phase variation shifts the signal between constructive and destructive superposition, and the dip was reproduced in all five repeats. Such phenomena are not uncommon in compact high-frequency antenna configurations, and they underscore the necessity of experimental validation alongside theoretical estimation.

The nonlinear relationship between isolation and SNR revealed in this study deepens the traditional understanding of this relationship. The SNR in the low isolation region is dominated by leakage noise, and the system performance in the optimal balance region is superior, whereas the SNR decreases owing to the beam offset when the isolation degree is too high. This finding shows that the design of the GB-SAR system is not a case where higher isolation is always better; rather, there exists an optimal balance area that must be considered. In this region, both the IM3 distortion caused by insufficient isolation and the synthetic aperture gain loss caused by excessive spatial separation can be effectively suppressed. This conclusion defines a clear target interval for parameter optimization in engineering practice and remedies the limitations of previous studies. Earlier works primarily concentrated on single-factor optimization while neglecting the system-coupling effect. Artifact severity is evaluated through a combination of three-level visual inspection and the quantitative artifact-to-background energy ratio (AER), as elaborated in Sections 2.4 and 3.4. The 3 dB AER boundary matches the isolation threshold of 74 dB, which transforms the original visual-only evaluation into a quantitatively verified classification framework while preserving intuitive grading labels. The IM3 relationship is analytically characterized based on the receiver third-order input intercept point and the 1-dB compression point, as demonstrated in formula (6) to formula (7). This approach derives the critical threshold from measurable nonlinear parameters. The exponential attenuation of artifact visibility beyond this threshold occurs inherently as leakage power falls outside the linear dynamic range of the receiver.

The establishment of 74 dB as the critical isolation threshold for eliminating range artifacts is an important finding of this study. This threshold is related to the specific parameters of the S-SAR M system, such as receiver linearity and noise floor; however, its underlying physical mechanism is universal. When the leakage signal is suppressed to a level where the resulting intermodulation products fall below the receiver noise floor, artifacts become invisible. The determination of this threshold provides a clear performance target for designing similar GB-SAR systems. We emphasize that this value should not be directly transplanted to systems with different center frequencies, antenna types, or receiver front-end designs. Artifacts below this value are bound to appear for the S-SAR M configuration, and artifact-free imaging can be achieved if the system reaches or exceeds this value, which has important engineering guiding value for ensuring the reliability of deformation monitoring data. For systems with different hardware parameters, engineers are advised to conduct a similar calibration procedure to derive their specific thresholds, using the methodology presented herein as a reference framework. formula (7) makes this calibration explicit, and the normalized margin $M=I_{\text{iso}}-I_{\text{iso},\text{th}}$, for which M at or above zero corresponds to artifact-free operation and values ranging from 0 dB to 6 dB belong to the optimal zone, generalizes the interval ranging from 74 dB to 80 dB to any platform.

Regarding alternative mitigation strategies, this study focused on antenna spatial parameter optimization because it represents a hardware-level solution that does not introduce additional computational burden or power consumption, which is essential for field-deployable GB-SAR systems. RF shielding can indeed improve isolation but often requires bulky metallic structures that compromise the compactness and portability required for slope monitoring in rough terrain. Digital cancellation techniques, while effective for direct leakage suppression, operate primarily on linear coupling mechanisms and are generally ineffective against the nonlinear IM3 distortion products that are the core concern of this study. Adaptive filtering in the signal processing chain can suppress artifacts to some extent but cannot recover the SNR degradation caused by front-end noise figure elevation. Therefore, the proposed dynamic spatial parameter design strategy offers a complementary and physically fundamental approach that addresses the root cause at the antenna architecture level. A concise comparison indicates that spatial parameter optimization achieves a favorable balance among isolation improvement, system compactness, and hardware simplicity, though its ultimate performance is bounded by the orbital length and beam width constraints of the specific platform. Table 5 summarizes this comparison quantitatively where measured values are available and as typical literature ranges otherwise; it also shows that spatial optimization is orthogonal to RF and digital techniques: it first drives leakage below the receiver nonlinear regime, after which shielding or cancellation can address any residual linear coupling, so the approaches can be cascaded rather than competing.

**Table 5:** Comparison of transmitter leakage and IM3 mitigation strategies.

Strategy	Typical isolation improvement	Action on nonlinear IM3	Added hardware / computational cost	Compatibility with spatial optimization
Antenna spatial parameter optimization (this study)	21–29 dB measured (53.3 to 74.6–82.9 dB)	Suppresses IM3 at its source by lowering leakage	None; parameter adjustment only, no added components	Baseline method
RF shielding / absorbing structures	Typically 10–20 dB	Partial; reduces the IM3 driving level	Bulky metallic/absorbing structure; reduces portability; no power cost	Complementary; can be cascaded
RF/analog and digital leakage cancellation	Typically 15–30 dB for linear leakage	Ineffective against nonlinear IM3 products	Additional RF channels and adaptive processing; moderate compute and power	Complementary for residual linear leakage
Adaptive digital filtering	No front-end isolation gain	Masks artifacts; cannot reverse noise-floor elevation	Software only; moderate compute	Post-processing complement only

The conclusion of this study is based on the S-SAR M system platform, and its specific threshold and optimal interval may vary with system parameters such as frequency, antenna type, and receiver design. The horizontal spacing values tested in this work ranged from 150 mm to 225 mm, which were selected based on the physical constraints of the S-SAR M orbital structure and the need to achieve meaningful isolation variation for the Ku-band horn antennas employed. Spacing below this range was found to produce isolation levels below 50 dB, where the system was entirely dominated by leakage noise without meaningful differentiation, and was therefore excluded from the reported experimental domain. Future research can further explore the universality of this rule in different GB-SAR systems and study how to maintain optimal long-term performance through parameter adjustment in dynamic deformation monitoring. Additionally, the proposed angle-priority plus spacing-coordination strategy provides directional guidance rather than a rigid mechanical prescription. The specific adjustment step size, mechanical tolerances, and phase error calibration procedures must be customized according to the orbital dimensions, antenna radiation patterns, and target distance requirements of each individual system. We view the present work as establishing a quantitative proof-of-concept and a methodological template that can be adapted by system engineers to their specific hardware constraints. Spatial parameter adjustment requires no additional components and thus incurs minimal marginal cost; the first-order performance-cost comparison in Table 5 confirms this advantage, while a full lifecycle cost-benefit analysis under field conditions remains a subject of future engineering study.

## 5. Conclusions

This study systematically elucidated the influence of transceiver isolation on the imaging quality of GB-SAR systems by employing a self-developed Ku-band S-SAR M system through rigorous parameter modeling and controlled experimentation. This study yields the following principal conclusions, which collectively furnish a coherent theoretical foundation and an actionable design framework for system optimization, with the explicit caveat that the numerical thresholds apply specifically to the S-SAR M hardware configuration.

First, the spatial configuration of the antennas, particularly the elevation angle, is paramount in determining the isolation performance. The experimental results demonstrated that increasing the angle from 0° to 60° enhanced isolation by an average of 56.3%, establishing angle as the dominant regulatory factor. The one-way ANOVA test yields F(3,12) = 97.9 with p less than 0.001. Furthermore, the horizontal spacing exhibits a significant synergistic effect at large angles, providing a critical degree of freedom for achieving high isolation within compact system dimensions. Engineers should note that these quantitative values were obtained for the S-SAR M system's horn antennas operating at 16.4 GHz, and systems with different antenna patterns or frequencies may exhibit different sensitivities.

Second, a sophisticated three-stage nonlinear coupling exists between the isolation and the system signal-to-noise ratio. In the low-isolation regime, the SNR is severely degraded by third-order intermodulation distortion noise generated by the transmitter leakage. An optimal balance region was identified between 74 and 80 dB, where IM3 was suppressed below the noise floor, and the SNR attained its maximum plateau. Conversely, pursuing isolation beyond 80 dB typically necessitates extreme spatial parameters that induce beam misalignment and synthetic aperture gain loss, ultimately leading to a decline in the SNR. This reveals a crucial design trade-off between suppressing leakage and maintaining optimal beam illumination. This tri-segmented relationship is described by a quadratic fit that achieves an R^2^ value of 0.92. This fit serves as the reduced empirical form of the mechanistic composite model presented in formula (10), which combines leakage-noise suppression with quadratic synthetic-aperture gain loss. The significance of this relationship is verified via ANOVA. The test returns F(3,12) = 18.5 with p less than 0.001, and larger-sample validation is planned for future work.

Third, a definitive critical isolation threshold of 74 dB was established for the S-SAR M system to achieve artifact-free imaging quality. When the isolation falls below this threshold, the power of the IM3 components exceeds the system noise floor, manifesting as coherent range-direction artifacts in the image. Attaining or surpassing this threshold effectively suppresses these artifacts, ensuring clean imaging, which is essential for reliable deformation monitoring. We reiterate that this threshold is system-specific and depends on the receiver noise floor, front-end linearity, and operating frequency. Generalization to other GB-SAR platforms should be performed through platform-specific calibration following the experimental methodology described in this paper. Equivalently, formula (7) and the normalized margin $M=I_{\text{iso}}-I_{\text{iso},\text{th}}$, where M at or above zero corresponds to artifact-free operation and values ranging from 0 dB to 6 dB belong to the optimal zone, provide an analytical, platform-independent procedure for this calibration.

In summary, this study quantified the chain linking spatial parameters, isolation, and the ultimate imaging quality. It advocates a dynamic design strategy prioritizing elevation angle control, complemented by spacing optimization, to navigate the identified optimal balance region. These findings provide practical guidelines for the hardware design of compact Ku-band GB-SAR systems of the studied class, addressing the challenge of reconciling compactness and high imaging fidelity. Statistical significance testing adopts ANOVA and Welch's t-tests, and all tests yield p less than 0.001. This testing approach together with a quantitative artifact energy metric is incorporated in this revision. Future research directions include larger repetition counts to obtain finer confidence intervals, validating the normalized-margin framework from formula (7) on GB-SAR platforms with different frequency bands, antenna types and receiver architectures, and testing the design guidelines under full outdoor operational conditions with environmental variability.
