# Supplementary material for: Research on the impact of transceiver isolation of GB-SAR on imaging quality
Source: PLoS One. 2026 Oct 29;21(10):e0360315. doi: 10.1371/journal.pone.0360315 (PMC13718967; doi:10.1371/journal.pone.0360315)
Supplement: Supplementary file 1 [file pone.0360315.s001.docx]

**Reviewer Report**

**General Assessment**

The manuscript entitled *“Research on the impact of transceiver isolation of GB-SAR on imaging quality”* investigates the influence of transmit–receive isolation on imaging performance in ground-based SAR systems using a self-developed Ku-band GB-SAR platform (S-SAR M). The topic is timely and relevant for compact GB-SAR system design. The study provides experimentally supported insights into the relationship between antenna spatial configuration, isolation, third-order intermodulation distortion (IM3), SNR degradation, and range artifacts.

The experimental methodology is generally sound, the results are clearly presented, and the conclusions are supported by the reported data. In particular, the identification of a system-specific isolation threshold and the observation of a nonlinear relationship between isolation and SNR represent valuable engineering contributions. Nevertheless, several issues should be addressed to strengthen scientific rigor, improve quantitative clarity, and better define the scope of generalization.

**Major Comments**

**1. Generalizability of the 74 dB Isolation Threshold**

The manuscript emphasizes 74 dB as a critical isolation threshold for artifact-free imaging. While this value is convincingly demonstrated for the S-SAR M system, it is inherently system-dependent.

The authors are encouraged to clearly and consistently state that this threshold depends on parameters such as receiver linearity, noise figure, operating frequency, and front-end design. A brief discussion or a simplified formulation expressing the isolation threshold as a function of parameters such as noise floor and third-order intercept point (IIP3) would significantly enhance the general applicability of the results.

**2. Quantification of Imaging Artifacts**

Artifact severity is currently categorized qualitatively as *strong*, *weak*, or *none* based on visual inspection of radar images.

For publication in a high-impact journal, the inclusion of at least one quantitative artifact metric (e.g., artifact-to-noise ratio, artifact energy relative to background, or an image contrast metric) would strengthen the objectivity of the analysis. If quantitative evaluation is not feasible, the authors should provide a clearer justification for the visual classification approach and explicitly discuss its limitations.

**3. IM3 Modeling and Receiver Nonlinearity**

The IM3 relationship is expressed in a proportional form (Eq. 5), which is physically reasonable but simplified.

The authors are encouraged to explicitly link this formulation to standard RF nonlinearity concepts such as the third-order intercept point (IIP3), or to clarify that the expression represents an approximate phenomenological model. A brief explanation of how receiver nonlinearity parameters influence IM3 generation and visibility would improve the technical depth of the manuscript.

**4. Comparison with Alternative Mitigation Strategies**

The manuscript briefly mentions RF shielding and digital cancellation techniques but does not provide a direct comparison.

A concise comparative discussion, or a summary table outlining the advantages, limitations, and implementation complexity of antenna spatial parameter optimization versus alternative mitigation strategies, would further enhance the engineering relevance and practical value of the study.

**Minor Comments**

1. Some sentences in the Introduction are overly long and could be simplified to improve readability.
2. Minor grammatical and typographical inconsistencies should be corrected throughout the manuscript.
3. The Conclusion section could be slightly shortened without loss of technical content.

**Recommendation**

**Major Revision**

The manuscript presents solid experimental work and meaningful engineering insights. Addressing the above comments—particularly those related to generalization, quantitative artifact assessment, and clarification of nonlinear modeling—would significantly strengthen the paper and make it suitable for publication.
