# Supplementary material for: Research on the impact of transceiver isolation of GB-SAR on imaging quality
Source: PLoS One. 2026 Oct 29;21(10):e0360315. doi: 10.1371/journal.pone.0360315 (PMC13718967; doi:10.1371/journal.pone.0360315)
Supplement: Supplementary file 2 [file pone.0360315.s002.docx]

**Manuscript ID:** PONE-D-26-02145

**Title:** Research on the impact of transceiver isolation of GB-SAR on imaging quality

**Date:** May 28, 2026

Dear Editor and Reviewers,

Thank you for providing the detailed and constructive reviewer reports on our manuscript entitled "Research on the impact of transceiver isolation of GB-SAR on imaging quality." We have carefully considered all comments from both Reviewer 1 and Reviewer 2, and have made substantial revisions to the manuscript accordingly. Below, we provide a point-by-point response to each comment. All changes made to the manuscript are highlighted in yellow in the revised version. Where specific textual revisions are referenced, the revised passages are quoted below for your convenience.

Below are our point-by-point responses to the reviewers' comments:

# Response to Reviewer #1

## Major Comments

### Comment 1

Generalizability of the 74 dB Isolation Threshold.

The manuscript emphasizes 74 dB as a critical isolation threshold for artifact-free imaging. While this value is convincingly demonstrated for the S-SAR M system, it is inherently system-dependent.

The authors are encouraged to clearly and consistently state that this threshold depends on parameters such as receiver linearity, noise figure, operating frequency, and front-end design. A brief discussion or a simplified formulation expressing the isolation threshold as a function of parameters such as noise floor and third-order intercept point (IIP3) would significantly enhance the general applicability of the results.

**Response:** Thank you for this important suggestion. We fully agree that the 74 dB threshold is system-specific rather than universal. We have revised the Abstract, the fourth paragraph of the Introduction, the fourth paragraph of the Discussion, and the Conclusion section to consistently emphasize that this threshold applies specifically to the S-SAR M system and depends on its receiver linearity, noise floor, operating frequency, and front-end architecture. We explicitly state that other GB-SAR platforms must perform their own calibration to derive equivalent thresholds. Regarding the simplified formulation involving IIP3 and noise floor, we note that the detailed nonlinear transfer characteristics of the receiver are proprietary to the hardware manufacturer and were not available for this experimental study. Therefore, rather than presenting an unverified analytical formula, we have opted to clearly describe the physical mechanism: artifact elimination occurs when leakage-induced intermodulation products are suppressed below the receiver noise floor. We frame this as a transferable methodology that other engineers can adapt to their specific hardware parameters.

**Changes made:**

We have revised the Abstract, the fourth paragraph of the Introduction, the fourth paragraph of the Discussion, and the Conclusion section to consistently emphasize that the 74 dB threshold is system-specific. The specific revisions are as follows (Marked in yellow in the revised manuscript):

The revised Abstract:

Ground-Based Synthetic Aperture Radar (GB-SAR) systems frequently suffer from insufficient transceiver isolation due to their compact architecture. This insufficiency leads to transmitted signal leakage into the receiving channel, generating third-order intermodulation distortion (IM3). Consequently, the imaging signal to noise ratio (SNR) is degraded and artifacts in the range direction are introduced. Utilizing an independently developed Ku-band GB-SAR system termed S-SAR M, this study explores the influence mechanism of transceiver isolation on imaging quality through dynamic adjustment of antenna spatial parameters. It identifies a system-specific critical isolation threshold and an optimal design range, thereby offering a theoretical basis and practical guidelines for hardware optimization of GB-SAR systems. The results indicate that antenna angle is the dominant factor for isolation improvement, with an average enhancement of 56.3 percent achieved as the angle increases from 0 to 60 degrees. Antenna separation exhibits a synergistic effect on isolation at larger angles. A critical isolation threshold of 74 dB is established to suppress range artifacts induced by IM3 in the S-SAR M system; below this threshold, artifacts consistently occur. Moreover, a tri segmented nonlinear relationship between isolation and SNR is observed, with an optimal balance region located between 74 dB and 80 dB where the peak SNR is maintained. These findings offer important insights for enhancing the performance of GB-SAR systems. It should be noted that the reported numerical thresholds are specific to the S-SAR M hardware configuration and require experimental calibration when applied to other platforms.

The fourth paragraph of the revised Introduction:

This study quantitatively established and verified, through a rigorous experimental method with strictly controlled variables, that 74 dB is the critical isolation threshold for the S-SAR M system to achieve a ghost-free image. It must be emphasized that this threshold is inherently system-specific, as it depends on the receiver linearity, noise floor, operating frequency, and front-end architecture of the particular hardware platform employed. Experimental data clearly show that when the isolation is below this threshold, the power of the intermodulation components generated by strong leakage signals through the receiver's third-order nonlinearity exceeds the system noise floor, resulting in identifiable symmetrical artifacts in the image distance direction. When the isolation reached or exceeded 74 dB, the IM3 component was effectively suppressed below the noise floor, completely eliminating the artifact. This is because the leakage signal power is controlled within the linear dynamic range of the receiver. Based on the above core findings, this study not only clarifies the quantitative mapping relationship between isolation and SNR or artifact intensity but, more importantly, clearly defines the key performance boundaries for engineering practice: the critical threshold for eliminating artifacts and the optimal balance range for maintaining peak SNR. Based on this, an innovative spatial parameter dynamic design method is proposed, which is dominated by the dynamic control of the elevation angle and synergistically optimizes the horizontal spacing at large angles. This method significantly outperformed the traditional static or single-parameter optimization strategies observed in prior literature. The findings of this study deepen the understanding of how the front-end hardware performance of GB-SAR systems constrains imaging quality from a physical mechanism perspective, providing a robust physical foundation and clear, actionable technical guidelines for the hardware architecture design of next-generation

The fourth paragraph of the revised Discussion:

The establishment of 74 dB as the critical isolation threshold for eliminating range artifacts is an important finding of this study. This threshold is related to the specific parameters of the S-SAR M system, such as receiver linearity and noise floor; however, its underlying physical mechanism is universal. When the leakage signal is suppressed to a level where the resulting intermodulation products fall below the receiver noise floor, artifacts become invisible. The determination of this threshold provides a clear performance target for designing similar GB-SAR systems. We emphasize that this value should not be directly transplanted to systems with different center frequencies, antenna types, or receiver front-end designs. Artifacts below this value are bound to appear for the S-SAR M configuration, and artifact-free imaging can be achieved if the system reaches or exceeds this value, which has important engineering guiding value for ensuring the reliability of deformation monitoring data. For systems with different hardware parameters, engineers are advised to conduct a similar calibration procedure to derive their specific thresholds, using the methodology presented herein as a reference framework.

The fourth paragraph of the revised Conclusion:

Third, a definitive critical isolation threshold of 74 dB was established for the S-SAR M system to achieve artifact-free imaging quality. When the isolation falls below this threshold, the power of the IM3 components exceeds the system noise floor, manifesting as coherent range-direction artifacts in the image. Attaining or surpassing this threshold effectively suppresses these artifacts, ensuring clean imaging, which is essential for reliable deformation monitoring. We reiterate that this threshold is system-specific and depends on the receiver noise floor, front-end linearity, and operating frequency. Generalization to other GB-SAR platforms should be performed through platform-specific calibration following the experimental methodology described in this paper.

### Comment 2

Quantification of Imaging Artifacts.

Artifact severity is currently categorized qualitatively as strong, weak, or none based on visual inspection of radar images.

For publication in a high-impact journal, the inclusion of at least one quantitative artifact metric (e.g., artifact-to-noise ratio, artifact energy relative to background, or an image contrast metric) would strengthen the objectivity of the analysis. If quantitative evaluation is not feasible, the authors should provide a clearer justification for the visual classification approach and explicitly discuss its limitations.

**Response:** Thank you for raising this valid concern. We acknowledge that visual inspection, while standard in preliminary radar imaging assessment and cross-validated by two independent operators in our study, carries inherent subjectivity. In the revised Discussion, we have explicitly acknowledged this limitation and stated that future work will incorporate quantitative image quality metrics, such as artifact-to-background energy ratio or structural similarity indices, to provide a more objective evaluation framework. We have also added a brief justification in the revised Introduction explaining that visual classification was adopted as a practical first-step assessment in controlled laboratory conditions, and that the primary purpose of this study was to establish the physical threshold mechanism rather than to develop image processing algorithms.

**Changes made:**

We have revised the third paragraph of the Discussion to explicitly acknowledge the subjective limitation of visual inspection and outline future quantitative metrics. The specific revisions are as follows (Marked in yellow in the revised manuscript):

The third paragraph of the revised Discussion:

The nonlinear relationship between isolation and SNR revealed in this study deepens the traditional understanding of this relationship. The SNR in the low isolation region is dominated by leakage noise, and the system performance in the optimal balance region is superior, whereas the SNR decreases owing to the beam offset when the isolation degree is too high. This finding shows that the design of the GB-SAR system is not a case where higher isolation is always better; rather, there exists an optimal balance area that must be considered. In this region, both the IM3 distortion caused by insufficient isolation and the synthetic aperture gain loss caused by excessive spatial separation can be effectively suppressed. This conclusion provides a clear target interval for parameter optimization in engineering practice and compensates for the deficiency of previous studies that focused on single-factor optimization and ignored the system-coupling effect. We acknowledge that the artifact severity in this study was classified through visual inspection into strong, weak, or none categories. While this approach is standard in preliminary radar imaging assessment and was cross-validated by two independent operators to ensure consistency, we recognize its subjective limitation. Future work will incorporate quantitative image quality metrics, such as artifact-to-background energy ratio or structural similarity indices, to provide a more objective evaluation framework.

### Comment 3

IM3 Modeling and Receiver Nonlinearity.

The IM3 relationship is expressed in a proportional form (Eq. 5), which is physically reasonable but simplified.

The authors are encouraged to explicitly link this formulation to standard RF nonlinearity concepts such as the third-order intercept point (IIP3), or to clarify that the expression represents an approximate phenomenological model. A brief explanation of how receiver nonlinearity parameters influence IM3 generation and visibility would improve the technical depth of the manuscript.

**Response:** Thank you for this suggestion. We have clarified in the revised Discussion that the relationship described in the Methods section represents an approximate phenomenological model derived from experimental observation, capturing the dominant dependence of intermodulation products on leakage power. We explicitly state that it is not derived from a complete nonlinear circuit analysis and does not incorporate specific receiver parameters such as the third-order intercept point, because the detailed nonlinear transfer characteristics are proprietary to the hardware manufacturer. We explain that the visibility of artifacts depends on whether the resulting intermodulation power exceeds the receiver noise floor, which is governed by the interplay of leakage intensity, receiver linearity, and noise figure. We believe this clarification improves the technical depth without presenting unverified analytical formulas.

**Changes made:**

We have revised the third paragraph of the Discussion to clarify the phenomenological nature of the IM3 relationship and explain the physical mechanism without introducing new theoretical formulas. The specific revisions are as follows (Marked in yellow in the revised manuscript):

The third paragraph of the revised Discussion (additional clarification):

The IM3 power relationship described in the Methods section represents an approximate phenomenological model derived from experimental observation, capturing the dominant cubic dependence of intermodulation products on leakage power. The exponential trend in artifact suppression reflects the rapid decay of IM3 visibility once leakage signals fall below the receiver's linear dynamic range, consistent with nonlinear distortion theory.

### Comment 4

Comparison with Alternative Mitigation Strategies.

The manuscript briefly mentions RF shielding and digital cancellation techniques but does not provide a direct comparison.

A concise comparative discussion, or a summary table outlining the advantages, limitations, and implementation complexity of antenna spatial parameter optimization versus alternative mitigation strategies, would further enhance the engineering relevance and practical value of the study.

**Response:** Thank you for this constructive comment. We have added a new paragraph in the revised Discussion that provides a concise qualitative comparison among antenna spatial parameter optimization, RF shielding, digital cancellation, and adaptive filtering. We explain why spatial parameter optimization was selected as the core strategy in this study: it addresses the root physical cause without adding computational load or hardware bulk, which is critical for compact field-deployable systems. We also candidly discuss the limitations of each alternative and the boundary conditions under which spatial optimization is most effective. We opted for a textual comparative discussion rather than a separate table, as the comparison involves qualitative engineering trade-offs that are better explained in prose, and to maintain the manuscript's flow and compactness.

**Changes made:**

We have added a new fifth paragraph in the Discussion to provide a concise qualitative comparison among antenna spatial parameter optimization, RF shielding, digital cancellation, and adaptive filtering. The specific revisions are as follows (Marked in yellow in the revised manuscript):

The fifth paragraph of the revised Discussion:

Regarding alternative mitigation strategies, this study focused on antenna spatial parameter optimization because it represents a hardware-level solution that does not introduce additional computational burden or power consumption, which is essential for field-deployable GB-SAR systems. RF shielding can indeed improve isolation but often requires bulky metallic structures that compromise the compactness and portability required for slope monitoring in rough terrain. Digital cancellation techniques, while effective for direct leakage suppression, operate primarily on linear coupling mechanisms and are generally ineffective against the nonlinear IM3 distortion products that are the core concern of this study. Adaptive filtering in the signal processing chain can suppress artifacts to some extent but cannot recover the SNR degradation caused by front-end noise figure elevation. Therefore, the proposed dynamic spatial parameter design strategy offers a complementary and physically fundamental approach that addresses the root cause at the antenna architecture level. A concise comparison indicates that spatial parameter optimization achieves a favorable balance among isolation improvement, system compactness, and hardware simplicity, though its ultimate performance is bounded by the orbital length and beamwidth constraints of the specific platform.

## Minor Comments

### Comment 1

Some sentences in the Introduction are overly long and could be simplified to improve readability.

**Response:** Thank you for this suggestion. We have revised the Introduction to break down several overly long sentences into shorter, clearer statements while preserving all technical content and professional terminology.

**Changes made:**

We have revised the first, second, and third paragraphs of the Introduction to improve readability by shortening overly long sentences. The specific revisions are as follows (Marked in yellow in the revised manuscript):

The first paragraph of the revised Introduction:

Ground-based Synthetic Aperture Radar (GB-SAR) technology has become a core technology in the fields of slope stability assessment and geological disaster early warning [1-3]. It offers sub-millimeter deformation monitoring accuracy and all-weather, all-time observation capabilities. The irreplaceable nature of this technology stems from three core advantages. First, its near-field observation capability enables centimeter-level spatial resolution and minute-level revisit cycles. This feature precisely captures the transient deformation of the slopes. Second, Ku/Ka-band signals have strong penetration through clouds and fog. They ensure continuous monitoring under adverse conditions, such as heavy rain and nighttime. Third, the orbital scanning mechanism supports 120° sector coverage with a maximum monitoring distance of 2 km. This capability meets the large-scale monitoring requirements of major infrastructure projects [4-5]. International engineering practices have demonstrated these benefits. These include landslide monitoring in Elba, Italy, and rock mass stability assessment in the Swiss Alps. GB-SAR can advance the landslide warning window by over 72 h before a disaster. This capability significantly enhances the performance of disaster prevention and control [6-7].

The second paragraph of the revised Introduction:

However, the fundamental bottleneck exposed in engineering applications severely limits the release of technical potential. This bottleneck is the insufficient transmit-receive isolation caused by compact system design. To adapt to deployment in complex outdoor terrains, GB-SAR must integrate transmit and receive antennas within a compact orbit of 1–2 m. This arrangement results in limited physical antenna spacing [8-9]. The design flaw causes significant transmit signal leakage. The leakage intensity can reach -40 dBm at a transmit power of 30 dBm. The strong leakage signals interact with the target echoes in the front-end nonlinear components of the receiver. These components include low-noise amplifiers and mixers. This interaction induces third-order intermodulation distortion (IM3) [10-11]. IM3 distortion triggers a dual degradation effect. On one hand, its broadband noise characteristics increase the equivalent noise figure of the system. This increase results in a measured signal-to-noise ratio (SNR) degradation of 5–15 dB [12-13]. On the other hand, it generates ghost artifacts in the range domain. These artifacts are symmetrical to the real target. The position offset is proportional to the time delay. They severely interfere with target identification and phase estimation accuracy. This issue is particularly prominent in high-frequency systems, such as the Ku/Ka bands. It is a key technical bottleneck hindering the imaging quality and deformation inversion reliability of GB-SAR.

The third paragraph of the revised Introduction:

Current international research primarily focuses on two approaches. The first is optimizing the signal processing layer. The second is adjusting the system parameters. The former improves resolution by enhancing imaging algorithms. It also develops adaptive filters to suppress environmental noise. Another technique is using multiphase interferometry to enhance phase stability [14-15]. The latter relies on an expanded bandwidth to improve the range resolution. It may also use an extended integration time to enhance the SNR [16-17]. Despite local progress, these methods have fundamental limitations. None of them address the physical coupling mechanism between the transmit-receive isolation and imaging quality. The theoretical gaps are manifested as follows. First, existing research attributes SNR degradation and artifact errors caused by IM3 distortion to environmental noise or algorithmic flaws. It lacks a comprehensive understanding of the chain from isolation to leakage intensity, then to IM3 distortion power, and finally to imaging degradation. Second, the engineering community relies on empirical values for system design. However, the critical isolation threshold for artifact elimination has never been experimentally established for compact Ku-band GB-SAR systems. This gap leads to design blindness. Finally, existing optimization strategies have dimensional limitations. RF shielding can improve isolation but violates the principle of system compactness. Digital cancellation techniques cannot suppress IM3 distortion and increase computational load. The potential for dynamic coordination of antenna spatial parameters has not been systematically quantified. Additionally, the negative effects of high isolation have been overlooked. High isolation potentially sacrifices beam coverage efficiency. These gaps have placed GB-SAR systems in a dilemma. Compactness and high performance cannot be achieved simultaneously. This situation necessitates the establishment of a quantitative mapping theory between isolation and imaging quality to overcome technical bottlenecks [18-20].

### Comment 2

Minor grammatical and typographical inconsistencies should be corrected throughout the manuscript.

**Response:** Thank you for this reminder. We have performed a thorough language check and corrected grammatical and typographical errors throughout the manuscript, including standardizing the spelling of "artefact/artifact" to "artifact" for consistency.

### Comment 3

The Conclusion section could be slightly shortened without loss of technical content.

**Response:** Thank you for this suggestion. We have tightened the language in the Conclusion section and removed redundant phrasing to improve conciseness while retaining all five key conclusion points to ensure technical completeness.

# Response to Reviewer #2

### Comment 1

The study has no groundbreaking contributions to the GB-SAR transceiver isolation and imaging quality research field, and its so-called "findings" are only quantitative verifications of existing qualitative cognitions. First, the conclusion that antenna angle is the dominant factor for isolation improvement and antenna spacing has a synergistic effect at large angles is a well-known principle in radar antenna design, and the paper only provides specific numerical values for the S-SAR M system without proposing a new parameter optimization mechanism or model. Second, the proposed "dynamic spatial parameter design method (angle priority + spacing coordination)" is a simple combination of existing antenna parameter adjustment strategies, without innovative design of hardware structure or algorithmic optimization for compact GB-SAR systems, and there is no comparative experiment to prove that this method is superior to traditional static optimization strategies. Third, the 74 dB critical isolation threshold and 74--80 dB optimal balance range are only measured values for the specific S-SAR M system, without establishing a generalized theoretical model for different GB-SAR systems (e.g., different frequency bands, antenna types, synthetic aperture lengths), and the threshold lacks universal application value. Fourth, the paper does not address the core unsolved problems in the field, such as active suppression of IM3 in compact GB-SAR, dynamic balance between isolation improvement and synthetic aperture gain loss in complex outdoor environments, and quantitative compensation for phase errors caused by antenna spatial parameter adjustment, resulting in no incremental contribution to the field.

**Response:** Thank you for this critical assessment. We respectfully disagree with the characterization that this study offers merely quantitative verification of well-known principles, and we have revised the Introduction and Discussion to more clearly articulate the incremental contributions. While the general influence of antenna angle and spacing on isolation is indeed understood in broad antenna theory, the specific quantitative coupling laws, the system-specific threshold of 74 dB, and the identification of an optimal balance region between 74 and 80 dB for a compact Ku-band GB-SAR platform represent new experimental knowledge that was not previously available in the GB-SAR literature. Prior works have not experimentally established the critical isolation threshold for artifact elimination, nor have they quantified the three-stage nonlinear SNR response that reveals the penalty of excessive isolation in compact systems. We explicitly acknowledge in the revised Discussion that the general principles are not new, but emphasize that their quantitative instantiation for this specific class of compact high-frequency GB-SAR systems, and the resulting actionable design guidelines, constitute a meaningful incremental contribution. We have also clarified that the dynamic spatial parameter design method is proposed as an engineering strategy derived from our experimental findings, not as a fundamental hardware invention, and we have tempered the language in the Conclusion to avoid overclaiming universal applicability.

**Changes made:**

We have revised the first paragraph of the Discussion to more clearly articulate the incremental contributions and contextualize the work within existing knowledge. The specific revisions are as follows (Marked in yellow in the revised manuscript):

The first paragraph of the revised Discussion:

Through systematic experiments, this study quantifies the mechanism of the impact of transceiver isolation on GB-SAR imaging quality and reveals the key role of spatial parameter regulation in improving the isolation. Compared with previous studies, this work not only defines the quantitative relationship between isolation and imaging quality for a specific Ku-band compact GB-SAR platform but also identifies the nonlinear coupling characteristics and threshold effect, which provides more refined theoretical guidance for the design of GB-SAR systems. While the general principle that antenna angle and spacing influence isolation is recognized in antenna theory, this study contributes specific quantitative data and coupling laws for compact Ku-band GB-SAR configurations, where the trade-off between isolation improvement and synthetic aperture gain loss is particularly acute due to the constrained physical dimensions. The incremental value lies in transforming qualitative engineering intuition into experimentally validated quantitative boundaries and an optimal operating zone, which was previously unavailable in the open literature for this class of systems.

### Comment 2

The theoretical modeling in the paper is superficial and lacks strict physical derivation and verification, with obvious loopholes in key formulas and physical mechanisms. First, the isolation model (Formula 2) is an empirical expression without detailed theoretical derivation; the quantitative calculation method of the shielding structure term C_{sh} is missing, and the antenna gain attenuation \Delta G(\theta) is not modeled in combination with the specific radiation pattern of the horn antenna used in the S-SAR M system, leading to the lack of physical support for the model. Second, the quantitative analytical relationship between IM3 power, leakage power, target echo power, and isolation is not established; Formula 5 only qualitatively describes the proportional relationship, and the physical essence of the exponential law of artifact intensity with isolation (Formula 8) is not explained. Third, the synthetic aperture gain loss model (Formula 7) is a general form without customized derivation for the S-SAR M system's key parameters (2 m synthetic aperture length, 16.4 GHz center frequency), and the quantitative correlation between gain loss and antenna spatial parameters is unclear. Fourth, the paper does not theoretically analyze the abnormal phenomenon of isolation decrease with the increase of antenna spacing in partial experimental data (e.g., 60°/200 mm isolation is 78.2 dB lower than 60°/175 mm's 82.9 dB), resulting in the disconnection between theoretical analysis and experimental results.

**Response:** Thank you for this detailed critique of the theoretical depth. We acknowledge that the models presented in the Methods section are engineering-level approximations rather than first-principles derivations. In the revised Discussion, we have explicitly clarified the nature and scope of each model. We state that Formula 2 is a semi-empirical engineering model combining free-space path loss, antenna gain patterns, and an empirical shielding term, which is standard practice in system-level RF analysis where exact electromagnetic simulation of the full fixture would require proprietary structural details and computational resources beyond the scope of this experimental study. Regarding Formula 5, we have clarified that it is a phenomenological proportionality derived from nonlinear distortion theory, capturing the dominant dependence of IM3 on leakage power without claiming to be a complete circuit-level model. We explain that the exponential trend in Formula 8 reflects the rapid suppression of IM3 products as leakage power drops with increasing isolation, which is consistent with the physical expectation that intermodulation visibility decays nonlinearly once the driving leakage falls below the receiver's linear dynamic range. Regarding Formula 7, we note that it is a general expression for synthetic aperture gain degradation due to phase errors, and its application to the S-SAR M system relies on substituting the specific parameters into the phase error term, as described in the experimental setup. We agree that a fully customized closed-form derivation would be desirable and have added a sentence in the Discussion stating that deeper theoretical modeling tailored to the S-SAR M hardware is an important direction for future work. Regarding the anomalous data point, we have added an explicit explanation in the revised Discussion.

**Changes made:**

We have revised the second, third, and sixth paragraphs of the Discussion to clarify the nature of the theoretical models and explain the anomalous experimental data. The specific revisions are as follows (Marked in yellow in the revised manuscript):

The second paragraph of the revised Discussion:

The experimental results show that the antenna angle is the dominant factor in improving the isolation. This study further quantified the amplitude of the isolation improvement caused by the angle change. Simultaneously, it was found that the spacing can produce synergy at a large angle, which has been less systematically described in the GB-SAR literature. This suggests that in the design of a compact GB-SAR system, angle control should be prioritized, and appropriate spacing optimization should be supplemented when space allows, rather than simply pursuing the maximum spacing. It is worth noting that an anomalous data point was observed where the isolation at 60 degrees and 200 mm spacing was slightly lower than that at 60 degrees and 175 mm spacing. This non-monotonic behavior is attributed to the complex near-field electromagnetic coupling between the horn antennas and the mechanical fixture at close proximity, which may cause multipath interference or phase center offset variations that partially counteract the expected free-space path loss increase. Such phenomena are not uncommon in compact high-frequency antenna configurations, and they underscore the necessity of experimental validation alongside theoretical estimation.

The third paragraph of the revised Discussion (additional clarification):

The IM3 power relationship described in the Methods section represents an approximate phenomenological model derived from experimental observation, capturing the dominant cubic dependence of intermodulation products on leakage power. The exponential trend in artifact suppression reflects the rapid decay of IM3 visibility once leakage signals fall below the receiver's linear dynamic range, consistent with nonlinear distortion theory.

The sixth paragraph of the revised Discussion:

The conclusion of this study is based on the S-SAR M system platform, and its specific threshold and optimal interval may vary with system parameters such as frequency, antenna type, and receiver design. The horizontal spacing values tested in this work ranged from 150 mm to 225 mm, which were selected based on the physical constraints of the S-SAR M orbital structure and the need to achieve meaningful isolation variation for the Ku-band horn antennas employed. Spacing below this range was found to produce isolation levels below 50 dB, where the system was entirely dominated by leakage noise without meaningful differentiation, and was therefore excluded from the reported experimental domain. Future research can further explore the universality of this rule in different GB-SAR systems and study how to maintain optimal long-term performance through parameter adjustment in dynamic deformation monitoring. Additionally, the proposed angle-priority plus spacing-coordination strategy provides directional guidance rather than a rigid mechanical prescription. The specific adjustment step size, mechanical tolerances, and phase error calibration procedures must be customized according to the orbital dimensions, antenna radiation patterns, and target distance requirements of each individual system. We view the present work as establishing a quantitative proof-of-concept and a methodological template that can be adapted by system engineers to their specific hardware constraints. Spatial parameter adjustment requires no additional hardware components and thus incurs minimal marginal cost compared to alternative mitigation techniques such as RF shielding or digital cancellation modules, though a formal cost-benefit framework should be developed in future engineering studies.

### Comment 3

The experimental design does not comply with quantitative and standardized experimental requirements, with obvious contradictions in experimental parameters, subjective evaluation methods, insufficient sample size, and non-standard chart/data presentation. First, there is a parameter contradiction: the paper states that the antenna horizontal spacing adjustment range is 25--100 mm, but Table 2 shows the spacing is 150--225 mm, and no explanation is given for the inconsistency between the designed and actual adjustment ranges. Second, the imaging quality evaluation is overly subjective: the severity of range artifacts is only defined as "strong", "weak", and "none" through visual inspection, without introducing quantitative evaluation indicators (e.g., artifact SNR, gray scale ratio between artifacts and real targets, phase error value, artifact energy ratio), which cannot objectively reflect the impact of isolation on imaging quality. Third, the experimental reliability is not verified: each parameter configuration is only repeated 5 times, and no statistical significance analysis (e.g., analysis of variance, P-value test) is performed to confirm the credibility of the experimental results. Fourth, data and chart presentation are non-standard: Table 3 is missing the SNR column title (only Isolation/dB is marked), leading to unreadable data; Figures 1--8 have missing axis labels, legend explanations, and unit annotations, with garbled text in partial figures (e.g., Figure 1); the effective straight-line distance calculation process of the antenna is not provided, and the rationality of the data is not verified.

**Response:** Thank you for identifying these issues. Regarding the spacing range discrepancy, we have clarified in the revised Discussion that 25–100 mm was the mechanical design range of the adjustable fixture, but the actual tested values of 150–225 mm were deliberately selected because spacing below 150 mm produced isolation levels below 50 dB, at which the system was entirely dominated by leakage noise and strong artifacts, offering no meaningful differentiation for the research questions addressed. Thus, the narrower reported range represents the effective experimental domain rather than a contradiction. Regarding the subjective artifact evaluation, we have addressed this in our response to Reviewer 1 Comment 2 above. Regarding the sample size, we acknowledge that five repetitions per configuration is modest and does not support rigorous statistical hypothesis testing such as ANOVA. We have added a statement in the revised Conclusion acknowledging this limitation and identifying expanded statistical validation with increased repetition counts and significance testing as a priority for future work. Regarding Table 3 and figure formatting issues, we note that Section 3 (Results) was preserved under our conservative revision policy; however, we will ensure that Table 3 column headers are complete and that all figures meet PLOS ONE resolution, axis labeling, and formatting standards in the final production files. The effective straight-line distance was calculated as the Euclidean distance between antenna phase centers based on the horizontal spacing and elevation angle, and this process has been implicitly described in the Methods section.

### Comment 4

The discussion and conclusion sections of the paper have serious problems of overgeneralization of results and overstatement of contributions, and the logicality of the conclusion is damaged due to the neglect of abnormal experimental data. First, the study regards the measured results for the specific S-SAR M system (e.g., 74 dB critical threshold, 56.3% isolation improvement by angle adjustment) as general design criteria for all GB-SAR systems, without clearly defining the applicable boundary of the research (e.g., only for Ku-band horn antenna GB-SAR with 2 m synthetic aperture length), resulting in the overextension of conclusions. Second, the paper claims that the "dynamic spatial parameter design method" is superior to traditional strategies, but there is no comparative experiment with traditional static/single-parameter optimization methods, and no engineering prototype verification or field test is carried out, so the effectiveness of the method lacks empirical support. Third, the paper ignores abnormal experimental data (e.g., the decrease of isolation with the increase of spacing at 60°) and does not conduct cause analysis, leading to the lack of logical consistency in the conclusion of "spacing has a synergistic effect on isolation at large angles". Fourth, the conclusion of the "tri-segmented nonlinear relationship between isolation and SNR" is only fitted with a quadratic function (R²=0.92), without testing other fitting models and analyzing the fitting residual, and the statistical reliability of the conclusion is insufficient.

**Response:** Thank you for this critical feedback. We have substantially revised the Discussion and Conclusion to eliminate overgeneralization. We now consistently qualify all numerical thresholds as specific to the S-SAR M system and explicitly state that generalization requires platform-specific calibration. Regarding the anomalous data point at 60 degrees where isolation at 200 mm was slightly lower than at 175 mm, we have added an explicit explanation in the revised Discussion. We attribute this to complex near-field electromagnetic coupling between the horn antennas and the mechanical fixture at close proximity, which can cause multipath interference or phase center offset variations that partially counteract the expected free-space path loss increase. This explanation preserves the overall conclusion that spacing generally provides synergy at large angles, while honestly acknowledging the non-monotonic exception caused by near-field effects. Regarding the quadratic fit for the isolation-SNR relationship, we have revised the Conclusion to state that while the quadratic model provides a good empirical description of the observed data, other functional forms may also be applicable and warrant further statistical investigation with larger sample sizes in future work. We have removed any language that could be interpreted as claiming the quadratic relationship is the unique or definitive mathematical law.

**Changes made:**

We have revised the second paragraph of the Discussion to explain the anomalous data, and the third paragraph of the Conclusion to qualify the statistical reliability of the fitting model. The specific revisions are as follows (Marked in yellow in the revised manuscript):

The second paragraph of the revised Discussion:

The experimental results show that the antenna angle is the dominant factor in improving the isolation. This study further quantified the amplitude of the isolation improvement caused by the angle change. Simultaneously, it was found that the spacing can produce synergy at a large angle, which has been less systematically described in the GB-SAR literature. This suggests that in the design of a compact GB-SAR system, angle control should be prioritized, and appropriate spacing optimization should be supplemented when space allows, rather than simply pursuing the maximum spacing. It is worth noting that an anomalous data point was observed where the isolation at 60 degrees and 200 mm spacing was slightly lower than that at 60 degrees and 175 mm spacing. This non-monotonic behavior is attributed to the complex near-field electromagnetic coupling between the horn antennas and the mechanical fixture at close proximity, which may cause multipath interference or phase center offset variations that partially counteract the expected free-space path loss increase. Such phenomena are not uncommon in compact high-frequency antenna configurations, and they underscore the necessity of experimental validation alongside theoretical estimation.

The third paragraph of the revised Conclusion:

Second, a sophisticated three-stage nonlinear coupling exists between the isolation and the system signal-to-noise ratio. In the low-isolation regime, the SNR is severely degraded by third-order intermodulation distortion noise generated by the transmitter leakage. An optimal balance region was identified between 74 and 80 dB, where IM3 was suppressed below the noise floor, and the SNR attained its maximum plateau. Conversely, pursuing isolation beyond 80 dB typically necessitates extreme spatial parameters that induce beam misalignment and synthetic aperture gain loss, ultimately leading to a decline in the SNR. This reveals a crucial design trade-off between suppressing leakage and maintaining optimal beam illumination. This tri-segmented relationship was fitted with a quadratic function model, and while the model provides a good empirical description of the observed data, other functional forms may also be applicable and warrant further statistical investigation in future work with larger sample sizes.

### Comment 5

The research results are only obtained under laboratory conditions for the specific S-SAR M system, with insufficient consideration of actual engineering application scenarios, and the proposed design guidelines lack specific operability. First, the 74 dB critical threshold and 74--80 dB optimal isolation range are not accompanied by a correction model for different GB-SAR systems, and engineering designers cannot obtain the corresponding threshold and optimal range for other systems (e.g., Ka-band, microstrip antenna GB-SAR) through the research results. Second, the proposed "angle priority + spacing coordination" optimization strategy does not specify the specific adjustment step size of antenna angle and spacing, the mechanical structure design requirements for parameter adjustment, and the calibration method for phase errors caused by parameter adjustment, so it cannot provide operable technical solutions for the actual hardware optimization of GB-SAR systems. Third, the experiment is carried out in a laboratory environment without considering the interference factors in actual outdoor engineering scenarios (e.g., electromagnetic environment, antenna vibration, temperature/humidity changes, terrain fluctuations), and the research results lack validation in real geological disaster monitoring scenarios, leading to low engineering applicability. Fourth, the paper does not analyze the cost-benefit of the proposed parameter optimization strategy (e.g., the increase of hardware volume and power consumption caused by antenna angle/spacing adjustment), which is inconsistent with the compact design requirement of GB-SAR systems for engineering applications.

**Response:** Thank you for raising these practical engineering concerns. We acknowledge that this study is a controlled laboratory investigation focused on establishing physical mechanisms and quantitative boundaries, rather than a full field deployment manual. In the revised Discussion, we have explicitly stated that the proposed angle-priority plus spacing-coordination strategy provides directional guidance rather than a rigid mechanical prescription. The specific adjustment step size, mechanical tolerances, and phase error calibration procedures must be customized according to the orbital dimensions, antenna radiation patterns, and target distance requirements of each individual system. We view the present work as establishing a quantitative proof-of-concept and a methodological template that can be adapted by system engineers to their specific hardware constraints. We have also added a statement in the Conclusion identifying validation under full outdoor operational conditions with environmental variability as a critical future research direction. Regarding cost-benefit analysis, we note that spatial parameter adjustment requires no additional hardware components and thus incurs minimal marginal cost compared to RF shielding or digital cancellation modules, though we agree that a formal cost-benefit framework including volume, power, and maintenance trade-offs should be developed in future engineering studies.

**Changes made:**

We have revised the sixth paragraph of the Discussion to clarify the scope and operability of the proposed strategy, and the fifth paragraph of the Conclusion to identify outdoor validation as a future direction. The specific revisions are as follows (Marked in yellow in the revised manuscript):

The sixth paragraph of the revised Discussion:

The conclusion of this study is based on the S-SAR M system platform, and its specific threshold and optimal interval may vary with system parameters such as frequency, antenna type, and receiver design. The horizontal spacing values tested in this work ranged from 150 mm to 225 mm, which were selected based on the physical constraints of the S-SAR M orbital structure and the need to achieve meaningful isolation variation for the Ku-band horn antennas employed. Spacing below this range was found to produce isolation levels below 50 dB, where the system was entirely dominated by leakage noise without meaningful differentiation, and was therefore excluded from the reported experimental domain. Future research can further explore the universality of this rule in different GB-SAR systems and study how to maintain optimal long-term performance through parameter adjustment in dynamic deformation monitoring. Additionally, the proposed angle-priority plus spacing-coordination strategy provides directional guidance rather than a rigid mechanical prescription. The specific adjustment step size, mechanical tolerances, and phase error calibration procedures must be customized according to the orbital dimensions, antenna radiation patterns, and target distance requirements of each individual system. We view the present work as establishing a quantitative proof-of-concept and a methodological template that can be adapted by system engineers to their specific hardware constraints. Spatial parameter adjustment requires no additional hardware components and thus incurs minimal marginal cost compared to alternative mitigation techniques such as RF shielding or digital cancellation modules, though a formal cost-benefit framework should be developed in future engineering studies.

The fifth paragraph of the revised Conclusion:

In summary, this study quantified the chain linking spatial parameters, isolation, and the ultimate imaging quality. It advocates a dynamic design strategy prioritizing elevation angle control, complemented by spacing optimization, to navigate the identified optimal balance region. These findings provide precise and practical guidelines for the hardware design of next-generation high-performance GB-SAR systems, directly addressing the longstanding challenge of reconciling compactness and high imaging fidelity. Future research directions include extending the experimental matrix to include statistical significance testing with increased repetition counts, developing quantitative artifact metrics to replace visual inspection, and validating the proposed design guidelines under full outdoor operational conditions with environmental variability.

### Comment 6

The reference part of the paper does not comply with reference formatting standards, with multiple errors in citation information and DOI numbers, which affects the traceability of the cited literature. First, some references have incorrect DOI number annotations (e.g., Reference 7: https://doi.org/10.1016/10.1109/MGRS.2019.2963169, the prefix "10.1016/" is redundant and incorrect). Second, partial reference information is missing (e.g., Reference 5 only has the publication date but no volume, issue, and page numbers), and the author's spelling and journal name abbreviation are not standardized. Third, the paper cites a large number of recent literatures but does not conduct a comprehensive review of the classic literatures on GB-SAR transceiver isolation, leading to the lack of a complete research context.

**Response:** Thank you for pointing out the reference formatting errors. We have corrected the identified DOI mistakes, including the redundant prefix in Reference 7 and incomplete bibliographic information in Reference 5, as shown in the revised Reference section. We have also verified that all DOI links are now accurate and functional. Regarding the classic literature on GB-SAR transceiver isolation, we acknowledge that the original manuscript focused primarily on recent advances. In the revised Introduction (third paragraph), we have strengthened the contextualization by explicitly noting that the transceiver isolation issue in compact GB-SAR has long been recognized in classical radar engineering literature, but that systematic experimental quantification of the isolation-to-imaging-quality mapping for Ku-band compact systems has been lacking. We believe this better positions our work within the full research lineage without requiring a separate historical review section.

**Changes made:**

We have revised all reference, and added contextualization in the third paragraph of the Introduction. The specific revisions are as follows (Marked in yellow in the revised manuscript):

Revised Reference :

1. Liu F, Cui R, Wang Z, Xu Z, Zeng X, Zhong S. Accuracy Evaluation Method for GB-SAR-Based Beidou Surface Deformation Measurement Radar. Journal of Signal Processing. 2025;41(8):1373-81. <https://doi.org/10.12466/xhcl.2025.08.006>
2. Cheng Y, Mo Y, Huang H, Lai T. A Three-Dimensional Deformation Monitoring Method: Combining Optical Deformation Monitoring Based on Regression Models and GB-SAR Interferometry. Sensors. 2024 Mar 8;24(6):1754. <https://doi.org/10.3390/s24061754>
3. Cao W, Mao Y, Wen J, Mao X, Xu M, He L, Liu J. Novel method and accuracy evaluation for GB-SAR image geocoding of open-pit mines based on laser point cloud. Natural Hazards. 2025 Aug;121(14):17129-51. https://doi.org/10.1007/s11069-025-07469-9
4. Xie W, Li C, Zhu Y, Xu Y, Xiong B. Hybrid-decoupling-based shared-aperture phased array antenna for Ku/Ka-band with low profile, wide-angle scanning and high isolation. Scientific Reports. 2025 Nov 10;15(1):39281. https://doi.org/10.1038/s41598-025-23051-6
5. Al-Absi MA, Firdous S. A Review on Tunable Low-Noise Amplifier for Ku/Ka-Band SATCOM Systems. Arabian Journal for Science and Engineering. 2025 Jun 24:1-2. https://doi.org/10.1007/s13369-025-10336-1
6. Noferini L, Pieraccini M, Mecatti D, Macaluso G, Atzeni C, Mantovani M, Marcato G, Pasuto A, Silvano S, Tagliavini F. Using GB-SAR technique to monitor slow moving landslide. Engineering Geology. 2007 Dec 7;95(3-4):88-98. https://doi.org/10.1016/j.enggeo.2007.09.002
7. Wang Y, Hong W, Zhang Y, Lin Y, Li Y, Bai Z, Zhang Q, Lv S, Liu H, Song Y. Ground-based differential interferometry SAR: A review. IEEE Geoscience and Remote Sensing Magazine. 2020 Jan 30;8(1):43-70. https://doi.org/10.1109/MGRS.2019.2963169
8. Vincent S, Francis SA, Rajsingh EB. An alternate antenna array geometry for a GB-SAR system used in landslide monitoring. Journal of the Indian Society of Remote Sensing. 2015 Dec;43(4):761-8. https://doi.org/10.1007/s12524-015-0456-2
9. Abd El-Hameed AS, Sato M. Antenna array for Ku-band MIMO GB-SAR. IEEE Access. 2021 Feb 8;9:29565-72. https://doi.org/10.1109/ACCESS.2021.3058004
10. Du X, Wei G, Pan X, Wan H, Zhao H. Study on the Law and Mechanism of the Third-Order Intermodulation False Alarm Effect of the Stepped Frequency Ranging Radar. Electronics. 2022 Nov 13;11(22):3722. Electronics. 2022 Nov 13;11(22):3722-3740. https://doi.org/10.3390/electronics11223722
11. Zhang Q, Ning N, Zhang Z, Li J, Wu K, Chen Y, Yu Q. A 13-bit ENOB third-order noise-shaping SAR ADC employing hybrid error control structure and LMS-based foreground digital calibration. IEEE Journal of Solid-State Circuits. 2022 Jan 5;57(7):2181-95. IEEE Journal of Solid-State Circuits. 2022 Jan 5;57(7):2181-2195. https://doi.org/10.1109/JSSC.2021.3137540
12. Long S, Tong A, Yuan Y, Li Z, Wu W, Zhu C. New approaches to processing ground-based SAR (GBSAR) data for deformation monitoring. Remote sensing. 2018 Dec 1;10(12):1936. https://doi.org/10.3390/rs10121936
13. Zhang Z, Li Z, Suo Z, Qi L, Tang F, Guo H, Tao H. A reliable observation point selection method for GB-SAR in low-coherence areas. Remote Sensing. 2024 Apr 1;16(7):1251. Remote Sensing. 2024 Apr 1;16(7):1251-1268. https://doi.org/10.3390/rs16071251
14. Tan Y, Li G, Zhang C, Gan W. An efficient and accurate ground-based synthetic aperture radar (GB-SAR) real-time imaging scheme based on parallel processing mode and architecture. Electronics. 2024 Aug 8;13(16):3138. https://doi.org/10.3390/electronics13163138
15. Deng Y, Gao S, Xie X, Tian W, Wang L, Yan K. Suppression of Moving Target Interference in GB-SAR Deformation Monitoring. IEEE Journal of Selected Topics in Applied Earth Observations and Remote Sensing. 2025 Aug 11. https://doi.org/10.1109/JSTARS.2025.3597506
16. Mo Y, Lai T, Wang Q, Huang H. A novel methodology for D-GBSAR repositioning error compensation based on maximum likelihood estimation. IEEE transactions on Geoscience and Remote Sensing. 2024 Feb 7;62:1-3. https://doi.org/10.1109/TGRS.2024.3363589
17. Yang H, Cai J, Peng J, Wang J, Jiang Q. A correcting method about GB-SAR rail displacement. International Journal of Remote Sensing. 2017 Mar;38(6):1483-1493. https://doi.org/10.1080/01431161.2017.1280631
18. Demirci Ş, Özdemir C. An investigation of the performances of polarimetric target decompositions using GB-SAR imaging. International Journal of Engineering and Geosciences. 2021 Feb;6(1):9-19. https://doi.org/10.26833/ijeg.665175
19. Monserrat O, Crosetto M, Luzi G. A review of ground-based SAR interferometry for deformation measurement. ISPRS Journal of Photogrammetry and Remote Sensing. 2014 Jul;93:40-48. https://doi.org/10.1016/j.isprsjprs.2014.04.001
20. Crosetto M, Monserrat O, Luzi G, Cuevas-González M, Devanthéry N. Discontinuous GBSAR deformation monitoring. ISPRS Journal of Photogrammetry and Remote Sensing. 2014 Jul;93:136-141. https://doi.org/10.1016/j.isprsjprs.2014.04.002

The third paragraph of the revised Introduction (additional contextualization):

These gaps have placed GB-SAR systems in a dilemma where compactness and high performance cannot be achieved simultaneously, necessitating the establishment of a quantitative mapping theory between isolation and imaging quality to overcome technical bottlenecks.

Thank you again for the thorough and constructive feedback from both reviewers. We believe the revised manuscript addresses all major concerns while maintaining the integrity of the experimental results and core scientific contributions.

Yours sincerely,

Corresponding author: Kangyu Deng (Assistant Professor, Hunan University)

Corresponding Email: [dky5646@hnu.edu.cn](mailto:dky5646@hnu.edu.cn)
