# Supplementary material for: Research on the impact of transceiver isolation of GB-SAR on imaging quality
Source: PLoS One. 2026 Oct 29;21(10):e0360315. doi: 10.1371/journal.pone.0360315 (PMC13718967; doi:10.1371/journal.pone.0360315)
Supplement: Supplementary file 3 [file pone.0360315.s003.docx]

**Manuscript ID: PONE-D-26-02145R1**

**Title: Research on the impact of transceiver isolation of GB-SAR on imaging quality**

**Date: 3 Sep 2026**

Dear Editor and Reviewers,

We sincerely thank the Academic Editor and Reviewer #2 for the careful fourth-round assessment, and Reviewer #3 for the positive recommendation. In this revision we have addressed every point raised by Reviewer #2 with substantive technical content rather than deferrals: the isolation model is now quantitatively calibrated against the measured horn pattern and geometry; the IM3 threshold is derived analytically from receiver nonlinearity parameters (IIP3 and the 1-dB compression point); the three-stage isolation–SNR relationship now has a mechanistic derivation; the 60° anomaly is analyzed quantitatively from electromagnetic first principles; statistical significance testing (ANOVA and Welch’s t-tests) and a quantitative artifact energy metric have been added; a normalized isolation-margin framework and a quantitative comparison table of mitigation strategies are provided; and the reference list, terminology, formula symbols and headings have been corrected. Two manuscript files are provided: a clean “Revised Manuscript” and a “Revised Manuscript with Track Changes” in which all additions are marked in red and deletions in red strikethrough. Our point-by-point responses follow; reviewer comments are reproduced in plain text and the corresponding revised passages are quoted in italics.

**Response to Reviewer #2**

**First: Theoretical modeling lacks rigorous physical derivation and reproducibility**

**Comment 1.** Eq.2 isolation model is a semi-empirical fitting formula: no quantitative calculation method is provided for the shielding structure term C_sh, and the gain attenuation term ΔG(θ) is not calibrated against the actual radiation pattern of the horn antenna used in the S-SAR M system.

**Response:** We agree, and Eq.2 is now fully calibrated rather than adjusted visually. The co-polar H-plane radiation pattern of the deployed horn was measured in 1° rotation steps to obtain ΔG(θ) for both antennas; C_sh is identified analytically at θ = 0° (where ΔG(0) = 0); and the net angular term was obtained by least-squares calibration against all 16 measured configurations. The calibrated model reproduces the measurements with an RMSE of 1.8 dB (1.4 dB excluding the single anomalous configuration), which establishes reproducibility.

**Changes made:**

*“Methods 2.1 now states: “The co-polar H-plane radiation pattern of the deployed horn was measured in 1° rotation steps, from which the off-boresight gain attenuation ΔG(θ) was obtained for both antennas. The shielding term C_sh is identified at θ = 0°, where ΔG(0)=0: it equals the mean over the four θ = 0° configurations of [I_iso(d,0)−20log10(4πd/λ)] plus the two boresight gains G_tx(0)+G_rx(0). The directly measurable intercept K = C_sh−G_tx(0)−G_rx(0) was determined as 11.3 ± 1.6 dB (mean ± standard deviation) … the net angular term G_a(θ) = ΔG(θ)+ΔC_sh(θ), which least-squares calibration gives as 22.2 dB, 20.5 dB and 19.2 dB at 30°, 45° and 60°. With these values, Eq.2 predicts all 16 measured isolation values with an RMSE of 1.8 dB (1.4 dB when the single anomalous configuration discussed in Section 3.1 is excluded), demonstrating that the model is physically grounded and independently reproducible.”*

**Comment 2.** Eq.5 third-order intermodulation power relationship remains phenomenological; it does not incorporate standard receiver nonlinearity parameters (IIP3, 1-dB compression point) and cannot analytically explain the physical origin of the 74 dB critical threshold.

**Response:** Standard third-order distortion theory has now been introduced. For the product 2f_leak−f_echo the leakage tone appears twice, giving an input-referred IM3 power P_IM3 = 2P_leak + P_echo − 2IIP3 (new Eq.6); the 1-dB compression constraint P_1dB ≈ IIP3−9.6 dB is stated; and equating P_IM3 to the noise floor yields the analytical threshold (new Eq.7). This shows that the critical threshold is set by four measurable parameters (transmit power, echo level, noise floor, IIP3), and for the S-SAR M system (P_tx = 30 dBm) the measured 74 dB corresponds analytically to a maximum tolerable leakage of −44 dBm.

**Changes made:**

*“Methods 2.1 now includes Eq.6 “P_IM3 = 2P_leak + P_echo − 2IIP3”, the sentence “Linear operation additionally requires the leakage to remain below the receiver 1-dB compression point P_1dB, related to IIP3 by P_1dB ≈ IIP3−9.6 dB”, Eq.7 “I_iso,th = P_tx − (P_n + 2IIP3 − P_echo)/2”, and: “Eq.7 identifies the four receiver-level parameters that set the critical threshold … For the S-SAR M system (P_tx = 30 dBm), the measured 74 dB threshold corresponds to a maximum tolerable leakage of −44 dBm at the receive port. Eq.7 also defines the generalized extrapolation route: for any other GB-SAR platform, substituting its own four parameters produces its platform-specific threshold without transplanting the numerical value 74 dB.”*

**Comment 3.** The tri-segmented nonlinear relationship between isolation and SNR is supported only by a quadratic fit without physical mechanism derivation.

**Response:** The quadratic fit is now presented as the reduced empirical form of an explicit mechanistic composite model (new Eq.10): the monotonic leakage-noise term from Eq.4 is superposed with the quadratic synthetic-aperture gain-loss term from Eq.9, and their crossover analytically generates the observed peak.

**Changes made:**

*“Section 3.3 now states: “This quadratic envelope is the reduced empirical form of a mechanistic composite model rather than an arbitrary polynomial fit: in the low-isolation regime Eq.4 makes SNR rise monotonically as leakage is suppressed, whereas in the high-isolation regime Eq.9 imposes a synthetic-aperture gain loss that grows quadratically with beam offset, and their superposition gives” Eq.10 “SNR(I) ≈ SNR_max − 10log10[1+10^((I_b−I)/10)] − κ(I−I_0)_+² … The crossover of these two analytic mechanisms generates the observed peak, providing the physical derivation that underlies the quadratic fit.”*

**Comment 4.** The anomalous finding that isolation decreases as antenna spacing increases at 60° is merely attributed to near-field electromagnetic coupling, with no electromagnetic simulation or targeted verification experiment.

**Response:** A quantitative first-principles analysis has been added (Section 3.1 and Discussion). At 16.4 GHz (λ = 18.3 mm), the first Fresnel-zone radius at l = 300–450 mm is 52–64 mm, comparable to the absorbing panels and fixture; a 25 mm spacing step changes the round-trip coupling path by 50 mm ≈ 2.7λ, rotating the reflected-coupling phase by ≈17 rad between constructive and destructive superposition; the Fraunhofer distance of the 35–40 mm horn aperture is only 134–175 mm, so the horns are mutually in the far field while the structural coupling paths remain in the radiating near field. The dip was reproduced in all five repeats, confirming a systematic near-field multipath ripple (±2–5 dB) rather than random error.

**Changes made:**

*“Section 3.1 adds: “The single non-monotonic point at 60° (78.2 dB at 200 mm versus 82.9 dB at 175 mm) was examined quantitatively using electromagnetic first principles … the first Fresnel-zone radius at l = 300–450 mm is 52–64 mm … a 25 mm spacing increment changes the round-trip coupling path by 50 mm ≈ 2.7 wavelengths, rotating the phase of the fixture-reflected coupling component by approximately 17 rad … the dip was reproduced in all five repeats at this configuration. It therefore does not alter the statistically dominant angle trend established below.”*

**Second: Methodological flaws in experimental design and image quality evaluation**

**Comment 5.** The antenna horizontal spacing adjustable range in Methods (25–100 mm) contradicts the actual experimental range (150–225 mm) in the data tables, and the selection rationale is not documented in the formal Methods section.

**Response:** The contradiction is resolved. The fixture’s mechanical travel (25–250 mm) is now distinguished from the deliberately selected effective test domain (150–225 mm), and the pre-screening rationale is documented in Methods 2.2 itself, rather than only in Discussion.

**Changes made:**

*“Methods 2.2 now reads: “This device supports continuous adjustment of the horizontal spacing d over a mechanical travel of 25–250 mm and independent adjustment of the relative angle θ … from 0° to 60°,” and: “Preliminary scans over the full 25–250 mm mechanical travel showed that for d < 150 mm the isolation remained below 53 dB (below 50 dB for d < 100 mm), where the receiver was entirely dominated by direct-coupling leakage and configurations could not be differentiated. This sub-range was consequently excluded from the formal experiment, whose effective domain is d = 150–225 mm at four levels and θ = 0°, 30°, 45°, 60°, as listed in Table 2; the 25–250 mm mechanical travel and the 150–225 mm effective test domain are therefore distinct by design, not contradictory.”*

**Comment 6.** Imaging artifact evaluation relies entirely on visual three-level classification with no quantitative metric; two-operator cross-validation is insufficient.

**Response:** A quantitative, operator-independent artifact-to-background energy ratio (AER) has been added in Methods 2.4, with explicit thresholds calibrated against the visual labels; the two-operator protocol is retained and supplemented by Cohen’s κ agreement; group-level AER statistics are reported in Section 3.4.

**Changes made:**

*“Methods 2.4 adds: “an artifact-to-background energy ratio (AER) was defined as AER = 10log10(P_art/P_bg) … no artifacts correspond to AER < 3 dB, weak artifacts to 3–6 dB, and strong artifacts to AER > 6 dB. Two operators independently performed both the visual labeling and the AER calculation for every image; inter-operator agreement was strong (Cohen’s κ = 0.87).” Section 3.4 adds: “the mean AER is 7.6 ± 1.0 dB for the four strong-artifact configurations, 4.4 ± 0.9 dB for the four weak-artifact configurations, and 1.3 ± 0.8 dB for the eight artifact-free configurations. Every configuration with isolation at or above 74 dB has an AER below 3 dB, so the independently computed AER boundary coincides exactly with the 74 dB isolation threshold.”*

**Comment 7.** Only five repeated measurements per configuration; no statistical significance analysis (ANOVA/hypothesis testing), so the 74 dB threshold and 74–80 dB optimal zone cannot be confirmed robust.

**Response:** Statistical testing has been added throughout. A one-way ANOVA across the four angle groups (four configurations per group) gives F(3,12) = 97.9 for isolation and F(3,12) = 18.5 for SNR (both p < 0.001); the low-isolation zone SNR (35.35 ± 0.31 dB, n = 4) differs significantly from the optimal-zone SNR (38.89 ± 0.82 dB, n = 9) by Welch’s t = 11.3 (df = 11, p < 0.001). The statistical method is declared in Methods 2.2, and the high-isolation SNR decline is shown consistent with the independently derived 1.6–3.5 dB aperture-loss estimate rather than random fluctuation.

**Changes made:**

*“Methods 2.2 adds: “one-way analysis of variance (ANOVA) across angle groups together with Welch’s t-tests between isolation zones was performed.” Section 3.2 adds: “A one-way ANOVA across the four angle groups … shows that angle has a highly significant effect on both isolation (F(3,12) = 97.9, p < 0.001) and SNR (F(3,12) = 18.5, p < 0.001. The mean SNR of the low-isolation zone (35.35 ± 0.31 dB, n = 4) is significantly lower than that of the optimal balance zone (38.89 ± 0.82 dB, n = 9; Welch’s t = 11.3, df = 11, p < 0.001) …”*

**Third: Limited academic novelty and risk of over-generalized conclusions**

**Comment 8.** The finding (angle dominant, spacing synergistic at large angles) is a well-recognized principle; the paper provides only specific measured values for a custom platform and offers no novel mechanism, hardware architecture or quantitative correction algorithm.

**Response:** We have explicitly delineated the incremental contribution. The Introduction now states what antenna theory already establishes qualitatively and identifies the specific gap that this paper closes—the complete, experimentally quantified chain from isolation through leakage and IM3 to imaging degradation for compact Ku-band GB-SAR; the Discussion states that the contribution is the transformation of qualitative engineering intuition into experimentally validated quantitative boundaries plus a calibrated model and an analytical threshold, rather than a claim of a new physical law. Over-claimed language (“innovative method that significantly outperforms …”, “next-generation high-precision, high-reliability equipment”) has been removed or scoped to “compact Ku-band GB-SAR systems of the studied class”.

**Changes made:**

*“Introduction now adds: “Although antenna theory qualitatively establishes that orientation and spacing govern inter-antenna coupling, the complete chain from isolation through leakage power and IM3 to imaging degradation has never been experimentally quantified for compact Ku-band GB-SAR, which is the specific gap addressed by the present study.” The former claim is replaced by: “A direct comparison on the measured data shows that the conventional spacing-only strategy at zero angle cannot cross the artifact-free threshold, whereas the proposed angle-priority rule does.”*

**Comment 9.** The paper still presents 74 dB and 74–80 dB as central findings without providing a generalized correction framework or extrapolation method for different GB-SAR systems.

**Response:** A generalized framework is now provided. Eq.7 expresses the threshold from receiver parameters, and a normalized isolation margin M = I_iso − I_iso,th is introduced: artifact-free operation corresponds to M ≥ 0 and the optimal balance zone to 0 ≤ M ≤ 6 dB, with the S-SAR M interval 74–80 dB being one realization. A four-step transfer procedure for any new platform is stated in Discussion and Conclusions.

**Changes made:**

*“Discussion 4 now adds: “define the normalized isolation margin M = I_iso − I_iso,th … artifact-free operation corresponds to M ≥ 0 and the optimal balance zone to 0 ≤ M ≤ 6 dB … For a new platform, designers (i) measure P_tx, P_echo, the noise floor and IIP3; (ii) compute I_iso,th from Eq.7; (iii) apply angle-priority spatial adjustment until M ≥ 0; and (iv) retain a margin no larger than about 6 dB to avoid synthetic-aperture gain loss. This procedure transfers the methodology without transplanting the numerical value 74 dB.”*

**Comment 10.** The proposed ‘dynamic spatial parameter design method’ is only a qualitative priority rule with no hardware implementation or comparative experiments against static design.

**Response:** A direct, data-based comparison against the conventional static/single-parameter strategy has been added using the same hardware and protocol: at θ = 0°, spacing-only adjustment varies isolation only from 52.7 to 53.7 dB and never approaches the 74 dB threshold, whereas the angle-priority strategy reaches 74.6–82.9 dB at 60° (a measured 21–29 dB gain). The proposal is now deliberately described as a “spatial parameter design rule / heuristic priority rule” rather than an over-stated new method.

**Changes made:**

*“Section 3.1 adds: “With the angle fixed at 0°, varying spacing alone changes isolation only from 52.7 to 53.7 dB and never approaches the 74 dB artifact-free threshold; in contrast, the proposed angle-priority adjustment reaches 74.6–82.9 dB at θ = 60°, a measured 21–29 dB improvement over the best spacing-only configuration. Obtained on the same hardware under the same test protocol, this is the empirical evidence that the dynamic spatial strategy outperforms a static, single-parameter design.”*

**Fourth: Logical evasion in the Discussion**

**Comment 11.** Core concerns (IM3 analytical modeling, quantitative artifact evaluation, generalized framework) are repeatedly responded to with ‘proprietary/future work’ statements rather than substantive content.

**Response:** All three items are now delivered in the manuscript itself rather than deferred: (i) the IM3 model is analytical through IIP3 and the 1-dB compression point (Eqs 6–7); (ii) artifact evaluation is quantitative through AER with group statistics; and (iii) the generalized framework is the normalized-margin procedure. Correspondingly, the previous “future work will incorporate quantitative metrics / phenomenological model” sentences in Discussion have been replaced with statements of what is now included.

**Changes made:**

*“Discussion now states: “Artifact severity is now assessed by both the three-level visual inspection and a quantitative artifact-to-background energy ratio (AER, Sections 2.4 and 3.4) …” and “The IM3 relationship is now expressed analytically through the receiver third-order input intercept point and the 1-dB compression point (Eqs 6–7), which derives the critical threshold from measurable nonlinearity parameters …”. The Conclusions likewise note that statistical testing and the quantitative artifact metric are incorporated in this revision.”*

**Comment 12.** The newly added comparison with alternative mitigation strategies is only qualitative; there is no quantitative performance-cost comparison or compatibility analysis with RF shielding or digital cancellation.

**Response:** A new Table 5 provides a structured performance-cost comparison: measured isolation improvement for the proposed method (21–29 dB), typical literature ranges for RF shielding (10–20 dB), RF/digital cancellation (15–30 dB for linear leakage) and adaptive digital filtering (no front-end gain), their action on nonlinear IM3, added hardware/computational cost, and compatibility. An explicit orthogonality/cascading analysis is added.

**Changes made:**

*“New Table 5 “Comparison of transmitter leakage and IM3 mitigation strategies” is added, and Discussion states: “Table 5 summarizes this comparison quantitatively where measured values are available and as typical literature ranges otherwise; it also shows that spatial optimization is orthogonal to RF and digital techniques: it first drives leakage below the receiver nonlinear regime, after which shielding or cancellation can address any residual linear coupling, so the approaches can be cascaded rather than competing.”*

**Comment 13.** The paper fails to address quantitative calculation of synthetic aperture gain loss; it does not derive the magnitude of phase error induced by beam misalignment using the specific S-SAR M parameters; the claim remains qualitative.

**Response:** A quantitative derivation using S-SAR M parameters (λ = 18.3 mm, L = 2 m, test distance r = 24 m) has been added. The lateral phase-center offset ε = d·sinθ gives a maximum differential phase ΔΦ_max = (2π/λ)(εL/2r) spanning 1.3–2.8 rad across the tested configurations; after standard focusing removes the linear ramp, the residual RMS phase error is 0.6–0.9 rad; the Ruze relation G_syn/G_0 ≈ exp(−σ_φ²) then gives a 1.6–3.5 dB gain loss, quantitatively consistent with the measured 2.5 dB SNR decline from the 41.2 dB peak.

**Changes made:**

*“Methods 2.1 now adds: “The lateral phase-center offset is ε = d·sinθ and the maximum differential phase across the aperture before focusing is ΔΦ_max = (2π/λ)(εL/2r), which ranges from 1.3 rad at (30°, 175 mm) to 2.8 rad at (60°, 225 mm) … leaving a residual root-mean-square phase error σ_φ of 0.6–0.9 rad; by the Ruze relation G_syn/G_0 ≈ exp(−σ_φ²) this corresponds to a synthetic-aperture gain loss of 1.6–3.5 dB, quantitatively consistent with the measured 2.5 dB SNR decline from the 41.2 dB peak …”*

**Fifth: Academic standardization and text issues**

**Comment 14.** The reference list is inconsistent: some entries duplicate the journal name (e.g., refs 10, 11, 13); some lack volume/issue/pages; DOI formats do not comply.

**Response:** All flagged entries have been corrected: the duplicated journal-name segments in refs 12, 13 and 16 were removed; the capitalization of refs 15 and 19 was corrected; all DOIs are now in the uniform https://doi.org/... format; and three classic references (Stove 1992; Leva et al. 2003; Caduff et al. 2015) have been added as refs 21–23.

All issues related to the references have been revised: Firstly, three classic references (Stove 1992; Leva et al. 2003; Caduff et al. 2015) have been added as references 4, 5, and 12. Therefore, the duplicate journal names in the original references 10, 11, and 13, which are now references 12, 13, and 16, have been removed;Thirdly, the capitalization of refs 15 and 19 was corrected; all DOIs are now in the uniform https://doi.org/... format; Finally, the color of the DOI links for all references has also been changed to blue.

**Changes made:**

*“Corrected entries now read, for example: “Du X, et al. … Electronics. 2022 Nov 13;11(22):3722-3740. https://doi.org/10.3390/electronics11223722”; “Zhang Q, et al. … IEEE Journal of Solid-State Circuits. 2022 Jan;57(7):2181-2195. https://doi.org/10.1109/JSSC.2021.3137540”; “Zhang Z, et al. … Remote Sensing. 2024 Apr 1;16(7):1251-1268. https://doi.org/10.3390/rs16071251”; and the newly added references 4, 5, and 12 are also included in the reference list”*

**Comment 15.** Terminology: ‘artefact’ and ‘artifact’ are used inconsistently across the main text and figure captions.

**Response:** The manuscript now uses the American spelling ‘artifact’ consistently in the main text, section headings, tables and all figure captions (zero occurrences of ‘artefact’ remain); Figure 12 was redrawn so that its in-figure labels likewise read ‘artifacts’/‘Reduced artifacts’.

**Changes made:**

*“Sections 2.4 and 3.4 headings now read “Distance artifact assessment” and “Isolation threshold effect of imaging artifacts”; Figure 12 has been regenerated with unified American-spelling labels.”*

**Comment 16.** Formula symbol typographical errors (such as the erroneous P_Lx for P_tx) remain uncorrected.

**Response:** All equations and inline symbols have been re-inspected one by one. Eq.1 correctly reads I_iso = 10log10(P_tx/P_leak) with P_tx (transmit power), and no P_Lx symbol exists anywhere in the current manuscript; the new Eqs 6–7 and 10 were typeset with the same symbol convention. A rendered check of every equation confirms the symbols.

**Changes made:**

*“Eq.1 is confirmed as “I_iso = 10log10(P_tx/P_leak)”, where “P_tx represents the transmission power and P_leak represents the power leaked into the receiving channel.”*

**Comment 17.** Introduction revisions are limited to splitting long sentences; classic GB-SAR transceiver isolation literature is not incorporated, the research lineage review is superficial, and the incremental contribution relative to established studies is not delineated.

**Response:** Three verifiable classic works have been incorporated: Stove (1992), the classic analysis of linear FMCW radar that establishes transmitter-to-receiver leakage as the sensitivity bottleneck of continuous-wave radar [12]; Leva et al. (2003), a foundational ground-based SAR interferometer landslide study [4]; and Caduff et al. (2015), the authoritative review of terrestrial radar interferometry [5]. The Introduction now cites these at the appropriate lineage points and explicitly delineates the present incremental contribution (see also our response to Comment 8).

**Changes made:**

*“Introduction now reads: “Insufficient isolation between co-located transmit and receive channels has been recognized as the fundamental sensitivity bottleneck of continuous-wave radar since the classic analysis of linear FMCW systems [12]”; “ground-based SAR interferometry has since matured into a well-documented geodetic technique for surface deformation monitoring [4-5]”; and the gap statement quoted in our response to Comment 8.”*

**Reviewer assessment question — Data availability (marked “No”)**

**Comment 18.** In the assessment questions, the availability of all data underlying the findings was marked “No”.

**Response:** All quantitative data underlying every conclusion in the manuscript are now explicitly stated to be contained in the manuscript itself: the complete 16-configuration isolation matrix is in Table 2, the corresponding SNR measurements in Table 3, and the artifact classifications in Table 4; all new statistics (ANOVA, Welch’s t-tests and AER group means) are computed directly from these tabulated values. A formal Data Availability statement has been added immediately before the References.

**Changes made:**

*“The manuscript now includes the heading “Data Availability” followed by: “All data underlying the findings of this study are included within the manuscript.”*

**Response to Reviewer #3**

We are grateful to Reviewer #3 for confirming that all prior concerns have been addressed and for the recommendation to publish. No further changes were requested by this reviewer.

**Closing statement**

We thank the editor and reviewers again. In this revision every concern of Reviewer #2 has been met with added derivations, calibrated model parameters, statistical tests, a quantitative image metric, a transferable normalized-margin framework, a quantitative mitigation-strategy comparison, and full standardization of references, terminology and symbols. We hope the manuscript is now suitable for publication in PLOS ONE and are happy to provide any further clarification.

Yours sincerely,

Corresponding author: Kangyu Deng (Assistant Professor, Hunan University)

Corresponding Email: [dky5646@hnu.edu.cn](mailto:dky5646@hnu.edu.cn)
