# Supplementary material for: Research on the impact of transceiver isolation of GB-SAR on imaging quality
Source: PLoS One. 2026 Oct 29;21(10):e0360315. doi: 10.1371/journal.pone.0360315 (PMC13718967; doi:10.1371/journal.pone.0360315)
Supplement: Peer review history

# Peer review: Decision Letter 0

**DOI:** 10.1371/journal.pone.0360315.r001

**Authors:** Neng Ye

11 Feb 2026

Dear Dr. Deng,

Thank you for submitting your manuscript to PLOS ONE. After careful consideration, we feel that it has merit but does not fully meet PLOS ONE’s publication criteria as it currently stands. Therefore, we invite you to submit a revised version of the manuscript that addresses the points raised during the review process.

Please submit your revised manuscript by Mar 28 2026 11:59PM. If you will need more time than this to complete your revisions, please reply to this message or contact the journal office at plosone@plos.org. When you're ready to submit your revision, log on to https://www.editorialmanager.com/pone/ and select the 'Submissions Needing Revision' folder to locate your manuscript file.

- A letter that responds to each point raised by the academic editor and reviewer(s). You should upload this letter as a separate file labeled 'Response to Reviewers'.
- A marked-up copy of your manuscript that highlights changes made to the original version. You should upload this as a separate file labeled 'Revised Manuscript with Track Changes'.
- An unmarked version of your revised paper without tracked changes. You should upload this as a separate file labeled 'Manuscript'.

If you would like to make changes to your financial disclosure, please include your updated statement in your cover letter. Guidelines for resubmitting your figure files are available below the reviewer comments at the end of this letter.

If applicable, we recommend that you deposit your laboratory protocols in protocols.io to enhance the reproducibility of your results. Protocols.io assigns your protocol its own identifier (DOI) so that it can be cited independently in the future. For instructions see: https://journals.plos.org/plosone/s/submission-guidelines#loc-laboratory-protocols. Additionally, PLOS ONE offers an option for publishing peer-reviewed Lab Protocol articles, which describe protocols hosted on protocols.io. Read more information on sharing protocols at https://plos.org/protocols?utm_medium=editorial-email&utm_source=authorletters&utm_campaign=protocols.

We look forward to receiving your revised manuscript.

Kind regards,

Neng Ye

Academic Editor

PLOS One

Journal Requirements:

When submitting your revision, we need you to address these additional requirements.

1. Please ensure that your manuscript meets PLOS ONE's style requirements, including those for file naming. The PLOS ONE style templates can be found at

https://journals.plos.org/plosone/s/file?id=wjVg/PLOSOne_formatting_sample_main_body.pdf and

https://journals.plos.org/plosone/s/file?id=ba62/PLOSOne_formatting_sample_title_authors_affiliations.pdf

2. Please note that PLOS One has specific guidelines on code sharing for submissions in which author-generated code underpins the findings in the manuscript. In these cases, we expect all author-generated code to be made available without restrictions upon publication of the work. Please review our guidelines at https://journals.plos.org/plosone/s/materials-and-software-sharing#loc-sharing-code and ensure that your code is shared in a way that follows best practice and facilitates reproducibility and reuse.

3. In your Methods section, please provide additional information regarding the permits you obtained for the work. Please ensure you have included the full name of the authority that approved the field site access and, if no permits were required, a brief statement explaining why.

4. Thank you for stating the following financial disclosure:

“Key Science and Technology Project of Ministry of Emergency Management of the People’s Re-public of China (No.2024EMST141405); Key Laboratory of Radar Imaging and Microwave Pho-tonics (Nanjing University of Aeronautics and Astronautics), Ministry of Education (No.NJ20250007)”

Please state what role the funders took in the study.  If the funders had no role, please state: "The funders had no role in study design, data collection and analysis, decision to publish, or preparation of the manuscript."

If this statement is not correct you must amend it as needed.

Please include this amended Role of Funder statement in your cover letter; we will change the online submission form on your behalf.

5. We note that your Data Availability Statement is currently as follows: “All relevant data are within the manuscript and its Supporting Information files”

Please confirm at this time whether or not your submission contains all raw data required to replicate the results of your study. Authors must share the “minimal data set” for their submission. PLOS defines the minimal data set to consist of the data required to replicate all study findings reported in the article, as well as related metadata and methods (https://journals.plos.org/plosone/s/data-availability#loc-minimal-data-set-definition).

For example, authors should submit the following data:

- The values behind the means, standard deviations and other measures reported;

- The values used to build graphs;

- The points extracted from images for analysis.

Authors do not need to submit their entire data set if only a portion of the data was used in the reported study.

If your submission does not contain these data, please either upload them as Supporting Information files or deposit them to a stable, public repository and provide us with the relevant URLs, DOIs, or accession numbers. For a list of recommended repositories, please see https://journals.plos.org/plosone/s/recommended-repositories.

If there are ethical or legal restrictions on sharing a de-identified data set, please explain them in detail (e.g., data contain potentially sensitive information, data are owned by a third-party organization, etc.) and who has imposed them (e.g., an ethics committee). Please also provide contact information for a data access committee, ethics committee, or other institutional body to which data requests may be sent. If data are owned by a third party, please indicate how others may request data access.

6. If the reviewer comments include a recommendation to cite specific previously published works, please review and evaluate these publications to determine whether they are relevant and should be cited. There is no requirement to cite these works unless the editor has indicated otherwise.

Additional Editor Comments:

The paper exhibits some innovation, but still requires revisions to meet publication standards

[Note: HTML markup is below. Please do not edit.]

Reviewers' comments:

Reviewer's Responses to Questions

**Comments to the Author**

1. Is the manuscript technically sound, and do the data support the conclusions?

Reviewer #1: Yes

Reviewer #2: Yes

**********

2. Has the statistical analysis been performed appropriately and rigorously?

Reviewer #1: Yes

Reviewer #2: No

**********

3. Have the authors made all data underlying the findings in their manuscript fully available?

The PLOS Data policy

Reviewer #1: Yes

Reviewer #2: No

**********

4. Is the manuscript presented in an intelligible fashion and written in standard English?

Reviewer #1: Yes

Reviewer #2: Yes

**********

Reviewer #1: Reviewer Report

General Assessment

The manuscript entitled “Research on the impact of transceiver isolation of GB-SAR on imaging quality” investigates the influence of transmit–receive isolation on imaging performance in ground-based SAR systems using a self-developed Ku-band GB-SAR platform (S-SAR M). The topic is timely and relevant for compact GB-SAR system design. The study provides experimentally supported insights into the relationship between antenna spatial configuration, isolation, third-order intermodulation distortion (IM3), SNR degradation, and range artifacts.

The experimental methodology is generally sound, the results are clearly presented, and the conclusions are supported by the reported data. In particular, the identification of a system-specific isolation threshold and the observation of a nonlinear relationship between isolation and SNR represent valuable engineering contributions. Nevertheless, several issues should be addressed to strengthen scientific rigor, improve quantitative clarity, and better define the scope of generalization.

Major Comments

1. Generalizability of the 74 dB Isolation Threshold

The manuscript emphasizes 74 dB as a critical isolation threshold for artifact-free imaging. While this value is convincingly demonstrated for the S-SAR M system, it is inherently system-dependent.

The authors are encouraged to clearly and consistently state that this threshold depends on parameters such as receiver linearity, noise figure, operating frequency, and front-end design. A brief discussion or a simplified formulation expressing the isolation threshold as a function of parameters such as noise floor and third-order intercept point (IIP3) would significantly enhance the general applicability of the results.

2. Quantification of Imaging Artifacts

Artifact severity is currently categorized qualitatively as strong, weak, or none based on visual inspection of radar images.

For publication in a high-impact journal, the inclusion of at least one quantitative artifact metric (e.g., artifact-to-noise ratio, artifact energy relative to background, or an image contrast metric) would strengthen the objectivity of the analysis. If quantitative evaluation is not feasible, the authors should provide a clearer justification for the visual classification approach and explicitly discuss its limitations.

3. IM3 Modeling and Receiver Nonlinearity

The IM3 relationship is expressed in a proportional form (Eq. 5), which is physically reasonable but simplified.

The authors are encouraged to explicitly link this formulation to standard RF nonlinearity concepts such as the third-order intercept point (IIP3), or to clarify that the expression represents an approximate phenomenological model. A brief explanation of how receiver nonlinearity parameters influence IM3 generation and visibility would improve the technical depth of the manuscript.

4. Comparison with Alternative Mitigation Strategies

The manuscript briefly mentions RF shielding and digital cancellation techniques but does not provide a direct comparison.

A concise comparative discussion, or a summary table outlining the advantages, limitations, and implementation complexity of antenna spatial parameter optimization versus alternative mitigation strategies, would further enhance the engineering relevance and practical value of the study.

Minor Comments

1. Some sentences in the Introduction are overly long and could be simplified to improve readability.

2. Minor grammatical and typographical inconsistencies should be corrected throughout the manuscript.

3. The Conclusion section could be slightly shortened without loss of technical content.

Reviewer #2: This paper investigates the impact of transceiver isolation on GB-SAR imaging quality using a self-developed Ku-band S-SAR M system, but it fails to meet the academic publication standards of PLOS One in multiple critical aspects. The most severe flaw is the severe lack of originality and innovation; the study only quantitatively verifies well-recognized phenomena in the existing GB-SAR field without proposing substantive new theories, methods, or strategies. In addition, there are obvious defects in theoretical analysis, experimental design, data presentation, and conclusion generalization, and the research contributions are overstated with insufficient engineering rigor and practical value.

1. The study has no groundbreaking contributions to the GB-SAR transceiver isolation and imaging quality research field, and its so-called "findings" are only quantitative verifications of existing qualitative cognitions. First, the conclusion that antenna angle is the dominant factor for isolation improvement and antenna spacing has a synergistic effect at large angles is a well-known principle in radar antenna design, and the paper only provides specific numerical values for the S-SAR M system without proposing a new parameter optimization mechanism or model. Second, the proposed "dynamic spatial parameter design method (angle priority + spacing coordination)" is a simple combination of existing antenna parameter adjustment strategies, without innovative design of hardware structure or algorithmic optimization for compact GB-SAR systems, and there is no comparative experiment to prove that this method is superior to traditional static optimization strategies. Third, the 74 dB critical isolation threshold and 74–80 dB optimal balance range are only measured values for the specific S-SAR M system, without establishing a generalized theoretical model for different GB-SAR systems (e.g., different frequency bands, antenna types, synthetic aperture lengths), and the threshold lacks universal application value. Fourth, the paper does not address the core unsolved problems in the field, such as active suppression of IM3 in compact GB-SAR, dynamic balance between isolation improvement and synthetic aperture gain loss in complex outdoor environments, and quantitative compensation for phase errors caused by antenna spatial parameter adjustment, resulting in no incremental contribution to the field.

2. The theoretical modeling in the paper is superficial and lacks strict physical derivation and verification, with obvious loopholes in key formulas and physical mechanisms. First, the isolation model (Formula 2) is an empirical expression without detailed theoretical derivation; the quantitative calculation method of the shielding structure term C_{sh} is missing, and the antenna gain attenuation \Delta G(\theta) is not modeled in combination with the specific radiation pattern of the horn antenna used in the S-SAR M system, leading to the lack of physical support for the model. Second, the quantitative analytical relationship between IM3 power, leakage power, target echo power, and isolation is not established; Formula 5 only qualitatively describes the proportional relationship, and the physical essence of the exponential law of artifact intensity with isolation (Formula 8) is not explained. Third, the synthetic aperture gain loss model (Formula 7) is a general form without customized derivation for the S-SAR M system's key parameters (2 m synthetic aperture length, 16.4 GHz center frequency), and the quantitative correlation between gain loss and antenna spatial parameters is unclear. Fourth, the paper does not theoretically analyze the abnormal phenomenon of isolation decrease with the increase of antenna spacing in partial experimental data (e.g., 60°/200 mm isolation is 78.2 dB lower than 60°/175 mm's 82.9 dB), resulting in the disconnection between theoretical analysis and experimental results.

3. The experimental design does not comply with quantitative and standardized experimental requirements, with obvious contradictions in experimental parameters, subjective evaluation methods, insufficient sample size, and non-standard chart/data presentation. First, there is a parameter contradiction: the paper states that the antenna horizontal spacing adjustment range is 25–100 mm, but Table 2 shows the spacing is 150–225 mm, and no explanation is given for the inconsistency between the designed and actual adjustment ranges. Second, the imaging quality evaluation is overly subjective: the severity of range artifacts is only defined as "strong", "weak", and "none" through visual inspection, without introducing quantitative evaluation indicators (e.g., artifact SNR, gray scale ratio between artifacts and real targets, phase error value, artifact energy ratio), which cannot objectively reflect the impact of isolation on imaging quality. Third, the experimental reliability is not verified: each parameter configuration is only repeated 5 times, and no statistical significance analysis (e.g., analysis of variance, P-value test) is performed to confirm the credibility of the experimental results. Fourth, data and chart presentation are non-standard: Table 3 is missing the SNR column title (only Isolation/dB is marked), leading to unreadable data; Figures 1–8 have missing axis labels, legend explanations, and unit annotations, with garbled text in partial figures (e.g., Figure 1); the effective straight-line distance calculation process of the antenna is not provided, and the rationality of the data is not verified.

4. The discussion and conclusion sections of the paper have serious problems of overgeneralization of results and overstatement of contributions, and the logicality of the conclusion is damaged due to the neglect of abnormal experimental data. First, the study regards the measured results for the specific S-SAR M system (e.g., 74 dB critical threshold, 56.3% isolation improvement by angle adjustment) as general design criteria for all GB-SAR systems, without clearly defining the applicable boundary of the research (e.g., only for Ku-band horn antenna GB-SAR with 2 m synthetic aperture length), resulting in the overextension of conclusions. Second, the paper claims that the "dynamic spatial parameter design method" is superior to traditional strategies, but there is no comparative experiment with traditional static/single-parameter optimization methods, and no engineering prototype verification or field test is carried out, so the effectiveness of the method lacks empirical support. Third, the paper ignores abnormal experimental data (e.g., the decrease of isolation with the increase of spacing at 60°) and does not conduct cause analysis, leading to the lack of logical consistency in the conclusion of "spacing has a synergistic effect on isolation at large angles". Fourth, the conclusion of the "tri-segmented nonlinear relationship between isolation and SNR" is only fitted with a quadratic function (R²=0.92), without testing other fitting models and analyzing the fitting residual, and the statistical reliability of the conclusion is insufficient.

5. The research results are only obtained under laboratory conditions for the specific S-SAR M system, with insufficient consideration of actual engineering application scenarios, and the proposed design guidelines lack specific operability. First, the 74 dB critical threshold and 74–80 dB optimal isolation range are not accompanied by a correction model for different GB-SAR systems, and engineering designers cannot obtain the corresponding threshold and optimal range for other systems (e.g., Ka-band, microstrip antenna GB-SAR) through the research results. Second, the proposed "angle priority + spacing coordination" optimization strategy does not specify the specific adjustment step size of antenna angle and spacing, the mechanical structure design requirements for parameter adjustment, and the calibration method for phase errors caused by parameter adjustment, so it cannot provide operable technical solutions for the actual hardware optimization of GB-SAR systems. Third, the experiment is carried out in a laboratory environment without considering the interference factors in actual outdoor engineering scenarios (e.g., electromagnetic environment, antenna vibration, temperature/humidity changes, terrain fluctuations), and the research results lack validation in real geological disaster monitoring scenarios, leading to low engineering applicability. Fourth, the paper does not analyze the cost-benefit of the proposed parameter optimization strategy (e.g., the increase of hardware volume and power consumption caused by antenna angle/spacing adjustment), which is inconsistent with the compact design requirement of GB-SAR systems for engineering applications.

6. The reference part of the paper does not comply with reference formatting standards, with multiple errors in citation information and DOI numbers, which affects the traceability of the cited literature. First, some references have incorrect DOI number annotations (e.g., Reference 7: https://doi.org/10.1016/10.1109/MGRS.2019.2963169, the prefix "10.1016/" is redundant and incorrect). Second, partial reference information is missing (e.g., Reference 5 only has the publication date but no volume, issue, and page numbers), and the author's spelling and journal name abbreviation are not standardized. Third, the paper cites a large number of recent literatures but does not conduct a comprehensive review of the classic literatures on GB-SAR transceiver isolation, leading to the lack of a complete research context.

**********

what does this mean?). If published, this will include your full peer review and any attached files.

If you choose “no”, your identity will remain anonymous but your review may still be made public.

**Do you want your identity to be public for this peer review?**  For information about this choice, including consent withdrawal, please see our Privacy Policy

Reviewer #1: No

Reviewer #2: No

**********

[NOTE: If reviewer comments were submitted as an attachment file, they will be attached to this email and accessible via the submission site. Please log into your account, locate the manuscript record, and check for the action link "View Attachments". If this link does not appear, there are no attachment files.]

To ensure your figures meet our technical requirements, please review our figure guidelines: https://journals.plos.org/plosone/s/figures

You may also use PLOS’s free figure tool, NAAS, to help you prepare publication quality figures: https://journals.plos.org/plosone/s/figures#loc-tools-for-figure-preparation.

NAAS will assess whether your figures meet our technical requirements by comparing each figure against our figure specifications.

Attachment Submitted filename: Reviewer Report TO PLOS ONE.docx

# Author comment: Author response to Decision Letter 1

**DOI:** 10.1371/journal.pone.0360315.r002

2 Jul 2026

Dear Reviewer:

Thank you for your hard work. I have made the necessary revisions according to your suggestions. The response to the reviewer's comments can be found in the "Response to Reviewers.docx" file.

Attachment Submitted filename: Response to Reviewers.docx

# Peer review: Decision Letter 1

**DOI:** 10.1371/journal.pone.0360315.r003

**Authors:** Sultan Shoaib

20 Jul 2026

Dear Dr. Deng,

Thank you for submitting your manuscript to PLOS ONE. After careful consideration, we feel that it has merit but does not fully meet PLOS ONE’s publication criteria as it currently stands. Therefore, we invite you to submit a revised version of the manuscript that addresses the points raised during the review process.

Please submit your revised manuscript by Sep 03 2026 11:59PM. If you will need more time than this to complete your revisions, please reply to this message or contact the journal office at plosone@plos.org. When you're ready to submit your revision, log on to https://www.editorialmanager.com/pone/ and select the 'Submissions Needing Revision' folder to locate your manuscript file.

- A letter that responds to each point raised by the academic editor and reviewer(s). You should upload this letter as a separate file labeled 'Response to Reviewers'.
- A marked-up copy of your manuscript that highlights changes made to the original version. You should upload this as a separate file labeled 'Revised Manuscript with Track Changes'.
- An unmarked version of your revised paper without tracked changes. You should upload this as a separate file labeled 'Manuscript'.

If you would like to make changes to your financial disclosure, please include your updated statement in your cover letter. Guidelines for resubmitting your figure files are available below the reviewer comments at the end of this letter.

If applicable, we recommend that you deposit your laboratory protocols in protocols.io to enhance the reproducibility of your results. Protocols.io assigns your protocol its own identifier (DOI) so that it can be cited independently in the future. For instructions see: https://journals.plos.org/plosone/s/submission-guidelines#loc-laboratory-protocols. Additionally, PLOS ONE offers an option for publishing peer-reviewed Lab Protocol articles, which describe protocols hosted on protocols.io. Read more information on sharing protocols at https://plos.org/protocols?utm_medium=editorial-email&utm_source=authorletters&utm_campaign=protocols.

As the corresponding author, your ORCID iD is verified in the submission system and will appear in the published article. PLOS supports the use of ORCID, and we encourage all coauthors to register for an ORCID iD and use it as well. Please encourage your coauthors to verify their ORCID iD within the submission system before final acceptance, as unverified ORCID iDs will not appear in the published article. *Only*  the individual author can complete the verification step; PLOS staff *cannot*  verify ORCID iDs on behalf of authors.

We look forward to receiving your revised manuscript.

Kind regards,

Sultan Shoaib

Academic Editor

PLOS One

Journal Requirements:

If the reviewer comments include a recommendation to cite specific previously published works, please review and evaluate these publications to determine whether they are relevant and should be cited. There is no requirement to cite these works unless the editor has indicated otherwise.

Additional Editor Comments :

Please address all the comments by the Reviewer 2

[Note: HTML markup is below. Please do not edit.]

Reviewers' comments:

Reviewer's Responses to Questions

**Comments to the Author**

Reviewer #2: (No Response)

Reviewer #3: All comments have been addressed

**********

2. Is the manuscript technically sound, and do the data support the conclusions?

Reviewer #2: Partly

Reviewer #3: Yes

**********

3. Has the statistical analysis been performed appropriately and rigorously?

Reviewer #2: N/A

Reviewer #3: Yes

**********

4. Have the authors made all data underlying the findings in their manuscript fully available?

The PLOS Data policy

Reviewer #2: No

Reviewer #3: Yes

**********

5. Is the manuscript presented in an intelligible fashion and written in standard English?

Reviewer #2: Yes

Reviewer #3: Yes

**********

Reviewer #2: Although the authors have revised the manuscript and provided point-by-point responses to the reviewer comments, the revised version still contains fundamental deficiencies in theoretical rigor, experimental methodology, academic novelty, and logical argumentation. A number of critical scientific and technical concerns raised in the original review have not been substantively addressed, and several new issues emerge from the revision. The manuscript does not currently satisfy the technical and scholarly standards for publication. The specific shortcomings are detailed below.

First, the theoretical modeling work lacks rigorous physical derivation and reproducibility. The isolation model expressed in Equation 2 remains a semi-empirical formulation, with no quantitative calculation method provided for the shielding structure term C_{sh}, and no calibration of the gain attenuation term \Delta G(\theta) against the actual radiation pattern of the horn antenna deployed in the S-SAR M system. This makes the model neither physically grounded nor independently reproducible. The third-order intermodulation power relationship in Equation 5 is still a phenomenological proportional relation that does not integrate key receiver nonlinearity parameters such as IIP3 or 1 dB compression point, and thus cannot analytically explain the physical origin of the 74 dB critical threshold. The proposed tri-segmented nonlinear relationship between isolation and signal-to-noise ratio is only supported by a quadratic function fit without derivation of its underlying physical mechanism, representing an empirical data observation rather than a theoretically derived conclusion. In addition, the anomalous finding that isolation decreases as antenna spacing increases at 60° is merely attributed to near-field electromagnetic coupling, with no supplementary electromagnetic simulation or targeted verification experiment to support this explanation. This unvalidated interpretation weakens the credibility of the core conclusion that antenna spacing produces a synergistic isolation gain at large angles.

Second, the experimental design and image quality evaluation suffer from systematic methodological flaws. The adjustable range of antenna horizontal spacing stated in the Methods section (25–100 mm) contradicts the actual experimental range (150–225 mm) presented in the data tables. Although the authors explain in their response that smaller spacing values were excluded because they yielded no meaningful differentiation, this selection rationale is not clearly documented in the formal Methods section of the manuscript, leading to insufficient transparency of the experimental design. More importantly, the evaluation of imaging artifacts still relies entirely on visual classification into three levels. Despite the authors' acknowledgment of its subjective limitation, no quantitative metric (e.g., artifact-to-noise ratio, artifact-to-background energy ratio) is added to support the classification, and cross-validation by two operators is insufficient to eliminate subjective bias. This evaluation approach does not meet the quantitative standard expected for imaging quality research. Furthermore, only five repeated measurements are performed for each parameter configuration, with no statistical significance analysis (such as analysis of variance or hypothesis testing) to verify the robustness of the results. Therefore, the key conclusions regarding the 74 dB threshold and the 74–80 dB optimal balance zone cannot be confirmed to be free from interference by random measurement errors.

Third, the academic novelty of the work is very limited, and the conclusions still carry a risk of overgeneralization despite added caveats. The finding that antenna angle is the dominant factor for isolation improvement and that spacing has a synergistic effect at large angles is a well-recognized principle in antenna engineering. This work merely provides specific measured values for a particular custom hardware platform, without proposing any novel optimization mechanism, innovative hardware architecture, or quantitative correction algorithm. The core contribution is essentially a parameter calibration for a single dedicated system, which provides very limited incremental knowledge to the GB-SAR research community. Although the authors have added statements emphasizing that the 74 dB threshold is system-specific, they still present this numerical value and the 74–80 dB optimal range as the central findings throughout the manuscript, and do not establish a generalized correction framework or extrapolation method applicable to GB-SAR systems with different frequency bands, antenna types, or receiver architectures. As a result, the engineering guidance value of the results is strictly confined to one specific platform, and cannot support the overstated claim that the work provides actionable guidelines for next-generation high-performance GB-SAR hardware design. In addition, the so-called “dynamic spatial parameter design method” is no more than a qualitative priority rule for parameter adjustment, with no corresponding hardware implementation scheme or comparative experiments against traditional static design strategies. Its claimed superiority over conventional approaches is completely unsupported by empirical evidence.

Fourth, the discussion section and the response to reviewers exhibit logical evasion and insufficient argumentation. For the core scientific concerns raised by the reviewers—including analytical modeling of IM3 distortion, quantitative artifact evaluation, and a generalized theoretical framework—the authors mostly respond by noting that detailed hardware parameters are proprietary or that related investigations will be conducted in future work, rather than supplementing substantive technical content in this revision. The addition of limitation disclaimers does not fundamentally resolve the identified academic deficiencies. In the newly added comparison with alternative mitigation strategies, the authors only provide qualitative descriptions of the pros and cons of each method, without quantitative performance-cost comparison or analysis of the compatibility between spatial parameter optimization and other techniques such as RF shielding and digital cancellation. This results in insufficient depth of the discussion on engineering trade-offs. Furthermore, the authors fail to address the issue of quantitative calculation of synthetic aperture gain loss. They do not derive the magnitude of phase error induced by beam misalignment using the specific parameters of the S-SAR M system, so the key mechanism that “SNR degradation in the high-isolation region arises from synthetic aperture gain loss” remains a qualitative inference without quantitative supporting evidence.

Fifth, there are still notable deficiencies in academic standardization and textual presentation. The reference list remains inconsistent in formatting: some entries duplicate the journal name (e.g., references 10, 11 and 13), some lack complete volume, issue and page information, and the DOI formats do not fully comply with the journal's specifications despite partial corrections. Terminology unification is incomplete, with inconsistent use of “artefact” and “artifact” across the main text and figure captions, and some typographical errors in formula symbols (such as the erroneous P_{Lx} for P_{tx}) remain uncorrected. In addition, the revisions to the Introduction are limited to splitting overly long sentences; the authors do not incorporate more classic literature on GB-SAR transceiver isolation to enrich the research background, and the review of the research lineage in this field remains superficial, failing to clearly delineate the specific incremental contribution of this work relative to established studies.

Reviewer #3: Authors have revised the manuscript and all concerns and comments have been addressed. I recommend publication

**********

what does this mean?). If published, this will include your full peer review and any attached files.

If you choose “no”, your identity will remain anonymous but your review may still be made public.

**Do you want your identity to be public for this peer review?**  For information about this choice, including consent withdrawal, please see our Privacy Policy

Reviewer #2: No

Reviewer #3: No

**********

[NOTE: If reviewer comments were submitted as an attachment file, they will be attached to this email and accessible via the submission site. Please log into your account, locate the manuscript record, and check for the action link "View Attachments". If this link does not appear, there are no attachment files.]

To ensure your figures meet our technical requirements, please review our figure guidelines: https://journals.plos.org/plosone/s/figures

You may also use PLOS’s free figure tool, NAAS, to help you prepare publication quality figures: https://journals.plos.org/plosone/s/figures#loc-tools-for-figure-preparation.

NAAS will assess whether your figures meet our technical requirements by comparing each figure against our figure specifications.

# Author comment: Author response to Decision Letter 2

**DOI:** 10.1371/journal.pone.0360315.r004

3 Sep 2026

Dear reviewers:

We sincerely thank Reviewer #2 for the rigorous and constructive comments, and Reviewer #3 for the positive recommendation. All comments have been addressed with substantive revisions rather than deferrals: (1) the isolation model is now calibrated against the measured horn radiation pattern (RMSE 1.8 dB), and the 74 dB threshold is derived analytically from receiver nonlinearity parameters (IIP3 and the 1-dB compression point); (2) one-way ANOVA and Welch's t-tests (all p < 0.001) and a quantitative artifact-to-background energy ratio (AER) have been added; (3) the three-stage isolation–SNR relationship is given a mechanistic derivation, and the 60° anomaly is explained quantitatively by near-field Fresnel-zone analysis; (4) a normalized isolation-margin framework and a quantitative comparison of mitigation strategies (new Table 5) are provided; and (5) the references, terminology, formula symbols and headings have been fully standardized. A detailed point-by-point response, with the corresponding revised passages quoted, is provided in the attached "Response to Reviewers", and all changes are marked in red in the "Revised Manuscript with Track Changes". Thank you again for your time and valuable guidance.

Attachment Submitted filename: Response_to_Reviewers_auresp_2.docx

# Editor report: Decision Letter 2

**DOI:** 10.1371/journal.pone.0360315.r005

**Authors:** Sultan Shoaib

25 Sep 2026

<p>Research on the impact of transceiver isolation of GB-SAR on imaging quality

PONE-D-26-02145R2

Dear Authors,

We’re pleased to inform you that your manuscript has been judged scientifically suitable for publication and will be formally accepted for publication once it meets all outstanding technical requirements.

Within one week, you’ll receive an e-mail detailing the required amendments. When these have been addressed, you’ll receive a formal acceptance letter and your manuscript will be scheduled for publication.

An invoice will be generated when your article is formally accepted. Please note, if your institution has a publishing partnership with PLOS and your article meets the relevant criteria, all or part of your publication costs will be covered. Please make sure your user information is up-to-date by logging into Editorial Manager at Editorial Manager® and clicking the ‘Update My Information' link at the top of the page. For questions related to billing, please contact billing support.

If your institution or institutions have a press office, please notify them about your upcoming paper to help maximize its impact. If they’ll be preparing press materials, please inform our press team as soon as possible -- no later than 48 hours after receiving the formal acceptance. Your manuscript will remain under strict press embargo until 2 pm Eastern Time on the date of publication. For more information, please contact onepress@plos.org.

Kind regards,

Sultan Shoaib

Academic Editor

PLOS One

Additional Editor Comments (optional):

The authors have made substantial efforts to address the comments and revise the manuscript. The revised version has improved significantly in terms of clarity, presentation, and overall quality. The concerns raised in the previous review have been adequately addressed. I have no further major comments and believe that the manuscript is now suitable for publication.

Reviewers' comments:

# Editor report: Acceptance letter

**DOI:** 10.1371/journal.pone.0360315.r006

**Authors:** Sultan Shoaib

PONE-D-26-02145R2

PLOS One

Dear Dr. Deng,

I'm pleased to inform you that your manuscript has been deemed suitable for publication in PLOS One. Congratulations! Your manuscript is now being handed over to our production team.

At this stage, our production department will prepare your paper for publication. This includes ensuring the following:

* All references, tables, and figures are properly cited

* All relevant supporting information is included in the manuscript submission,

* There are no issues that prevent the paper from being properly typeset

You will receive further instructions from the production team, including instructions on how to review your proof when it is ready. Please keep in mind that we are working through a large volume of accepted articles, so please give us a few days to review your paper and let you know the next and final steps.

Lastly, if your institution or institutions have a press office, please let them know about your upcoming paper now to help maximize its impact. If they'll be preparing press materials, please inform our press team within the next 48 hours. Your manuscript will remain under strict press embargo until 2 pm Eastern Time on the date of publication. For more information, please contact onepress@plos.org.

You will receive an invoice from PLOS for your publication fee after your manuscript has reached the completed accept phase. If you receive an email requesting payment before acceptance or for any other service, this may be a phishing scheme. Learn how to identify phishing emails and protect your accounts at https://explore.plos.org/phishing.

If we can help with anything else, please email us at customercare@plos.org.

Thank you for submitting your work to PLOS One and supporting open access.

Kind regards,

PLOS One Editorial Office Staff

on behalf of

Dr. Sultan Shoaib

Academic Editor

PLOS One
